# Differentiating nontuberculous mycobacterium pulmonary disease from pulmonary tuberculosis through the analysis of the cavity features in CT images using radiomics

**DOI:** 10.1186/s12890-021-01766-2

**Published:** 2022-01-07

**Authors:** Qinghu Yan, Wuzhang Wang, Wenlong Zhao, Liping Zuo, Dongdong Wang, Xiangfei Chai, Jia Cui

**Affiliations:** 1 Department of Radiology, Shandong Public Health Clinical Center, Jinan, 250013 China; 2 Department of Radiology, Qilu Hospital of Shandong University, Jinan, 250012 China; 3 Huiying Medical Technology (Beijing) Co., Ltd, Beijing, 100192 China

**Keywords:** Radiomics, Cavity, CT, Nontuberculous mycobacteria, Pulmonary tuberculosis

## Abstract

**Objective:**

To differentiate nontuberculous mycobacteria (NTM) pulmonary diseases from pulmonary tuberculosis (PTB) by analyzing the CT radiomics features of their cavity.

**Methods:**

73 patients of NTM pulmonary diseases and 69 patients of PTB with the cavity in Shandong Province Chest Hospital and Qilu Hospital of Shandong University were retrospectively analyzed. 20 patients of NTM pulmonary diseases and 20 patients of PTB with the cavity in Jinan Infectious Disease Hospitall were collected for external validation of the model. 379 cavities as the region of interesting (ROI) from chest CT images were performed by 2 experienced radiologists. 80% of cavities were allocated to the training set and 20% to the validation set using a random number generated by a computer. 1409 radiomics features extracted from the Huiying Radcloud platform were used to analyze the two kinds of diseases' CT cavity characteristics. Feature selection was performed using analysis of variance (ANOVA) and least absolute shrinkage and selection operator (LASSO) methods, and six supervised learning classifiers (KNN, SVM, XGBoost, RF, LR, and DT models) were used to analyze the features.

**Results:**

29 optimal features were selected by the variance threshold method, K best method, and Lasso algorithm.and the ROC curve values are obtained. In the training set, the AUC values of the six models were all greater than 0.97, 95% CI were 0.95–1.00, the sensitivity was greater than 0.92, and the specificity was greater than 0.92. In the validation set, the AUC values of the six models were all greater than 0.84, 95% CI were 0.76–1.00, the sensitivity was greater than 0.79, and the specificity was greater than 0.79. In the external validation set, The AUC values of the six models were all greater than 0.84, LR classifier has the highest precision, recall and F1-score, which were 0.92, 0.94, 0.93.

**Conclusion:**

The radiomics features extracted from cavity on CT images can provide effective proof in distinguishing the NTM pulmonary disease from PTB, and the radiomics analysis shows a more accurate diagnosis than the radiologists. Among the six classifiers, LR classifier has the best performance in identifying two diseases.

**Supplementary Information:**

The online version contains supplementary material available at 10.1186/s12890-021-01766-2.

## Background

Globally, the incidence of nontuberculous mycobacteria (NTM) has been increasing rapidly in recent years [1, 2]. NTM, usually involving the lung, exhibits characteristics such as slow growth, long course, and high resistance to first-line anti-tuberculosis drugs, resulting in a low total care rate [3]. To improve the treatment effect, pretreatment diagnosis is very crucial. However, the CT features and the clinical manifestations of the NTM pulmonary disease are similar to those of pulmonary tuberculosis (PTB), so it is very difficult to identify these two diseases simply via CT or clinical features. At present, the main methods of distinguishing them include the culture of mycobacterium in sputum and the identification of the species. However, these methods are time-and resource-consuming and require higher laboratory standards, which cannot be provided in many local hospitals [4]. Moreover, even with a positive culture result, there is still a need for further identification of the species. Once being suspected of PTB with positive sputum AFB, a patient will be administered an empirical anti-TB medicine for treatment when the test is ongoing to identify the bacteria. This means that some patients may receive unnecessary treatment, which may consequently result in the risk of adverse drug reactions, with its accompanying unnecessary medical expenses [5]. Therefore, the early diagnosis of NTM pulmonary disease can improve the quality of patients’ life, and contribute to disease treatment. Some studies [6–9] have shown that there are usually changes in the imaging of NTM pulmonary disease. A Tomography (CT) scan can be used to identify the changes in the lung areas including cavity formation, parenchymal lesion, bronchiectasis, and tree-in-bud patterns [9, 10, 20], particularly the cavity, and the cavity is also a common imaging feature of chronic PTB [11, 12]. Unfortunately, the CT cavity images do not provide effective identification points in these two diseases. In recent years, radiomics, mining data features through high-throughput extraction have shown great potential in the diagnosis and differentiation of lung diseases, such as pulmonary nodules and early lung cancer [13, 14], and this may provide a feasible method of identifying the NTM pulmonary disease from PTB. In addition, the pathological difference in the cavity between the two diseases has been proven [15], which increases their possibility of identification. Thus, this study investigates the value of CT radiomics in the cavity analysis and identification of the NTM and PTB pulmonary diseases. To explore the significance of this technique in the differential diagnosis of the two diseases, select excellent classifiers, and provide a new and simple diagnostic method for clinical treatment.

## Materials and methods

### Patient information

The study retrospectively analyzed 89 patients with NTM pulmonary disease and other 100 cases of patients with PTB [16] in CT imaging in the Shandong Provincial Chest Hospital and Qilu Hospital of Shandong University from February 2013 to March 2018. In addition, 27 patients with NTM pulmonary disease and other 30 cases of patients with PTB [16] in CT imaging at The Jinan Infectious Disease Hospitall were collected for external validation of the model. The content of this study was approved by the ethics committee of Shandong Provincial Chest Hospital (Additional files 1, 2, 3). All patients with lung changes underwent a plain chest scan using Philips 64-slice spiral CT scanner before treatment, and the quality correction was met before the CT scan. The NTM pulmonary disease or PTB patient was laid in a supine position, and CT scanned from the tip of the lungs to the level of the thoracic cavity with the patient holding his breath. The CT scanning parameters were as follows: the diameter of the inspected detector, 64 × 0.625 mm; the rotation time, 0.5 s; the pitch, 1.375; the tube voltage, 120 kV; the tube current, 250 ~ 400 mA, which was modulated using an automatic tube current; and the thickness of the slice, 5 mm.

The inclusion criteria: (1) All cases of patients with NTM pulmonary disease who were diagnosed with sputum cultured twice and the strains were identified as the same pathogenic bacteria [12, 17]. All cases of patients with PTB who were identified by the new version of the tuberculosis diagnosis and tuberculosis classification standard issued by the China National Health Service Commission on November 9, 2017. The sputum smear of the same patient was positive for acid-fast bacilli at least once and was identified as *Mycobacterium tuberculosis* complex using the colloidal gold method. Also, clinical symptoms, laboratory examination, or imaging features suggest tuberculosis infection (2). The CT images (lung window) indicated the existence of a more than 3 mm cavity. (3) Corresponding anti-NTM or -PTB treatment was effective. (4) All images satisfied the analytical criteria for radiomics. Exclusion criteria: (1) the presence of lesions without cavities as depicted by the CT image (2) Some factors might influence the accuracy of the analysis including the CT images with motion artifacts, poor image quality, massive differences in scanning parameters, and inconsistent slice thicknesses. (3) The bullae and other cavities without a wall. (4) cases in which corresponding treatments had no effect. (5) The clinical data showed that the patient had the possibility of mixed infection. (6) The outer wall is difficult to be delineated because it was covered by the surrounding opacities or consolidation. After screening, 23 patients with NTM pulmonary disease and 41 patients with PTB were excluded, thus leaving a total of 73 patients with NTM pulmonary disease, including 47 males and 26 females, with an average age of 57.3 ± 14.0 years (ranged from 23- 86 years) and 69 patients with PTB, including 48 males and 21 females; mean age 45.7 ± 17.9 years (ranged from 17–90 years) to participate in this study. The external validation model had 20 patients with NTM pulmonary diseases (15 men and 5 women; mean age, 52.2 ± 16.2 years; range, 17–72 years), and 20 patients with PTB (12 men and 8 women; mean age, 43.8 ± 17.7 years; range, 19–76 years), with the cavity.

### Characterization of lesions

**Fig. 1:**
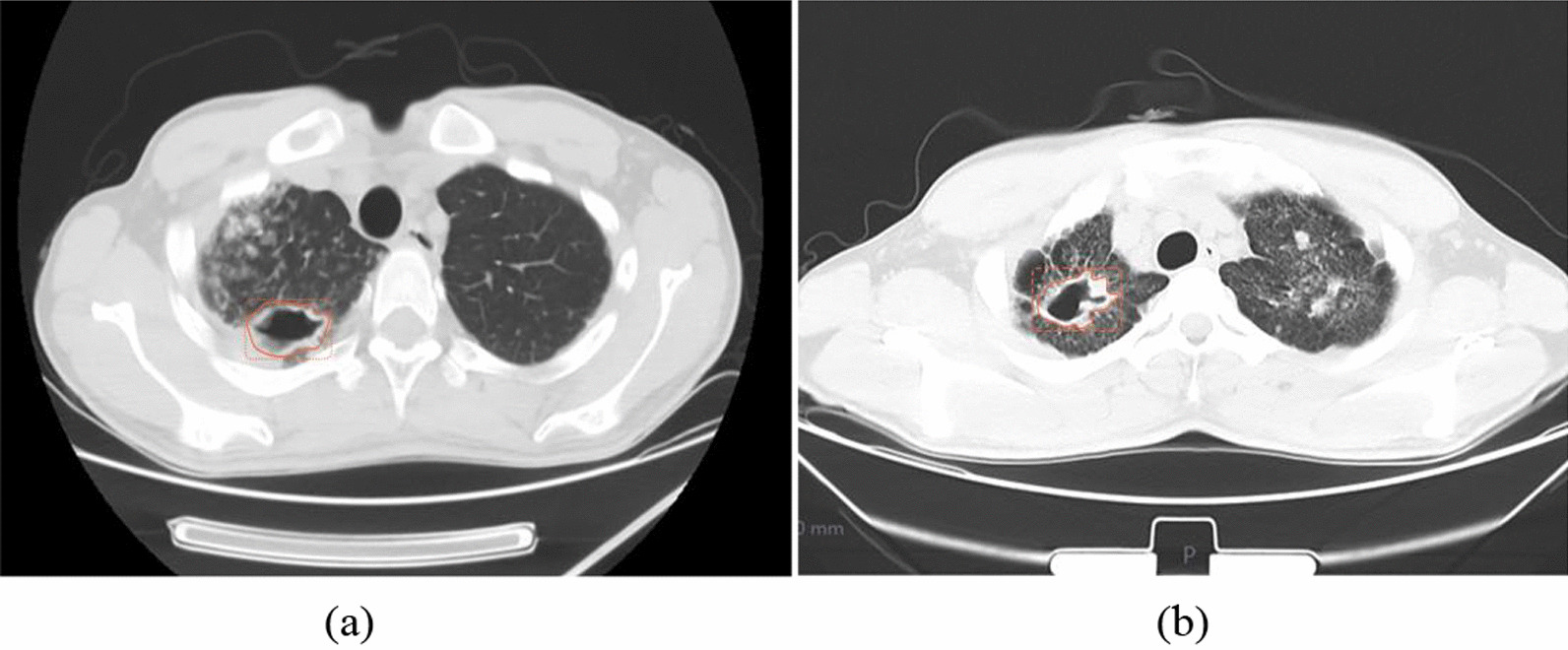
**a** A patient (male, 54 years old) with a cavity of NTM pulmonary disease in the right superior lobe. Outline along the outer edge of the cavity and include the cavity wall. **b** A patient (male, 65 years old) with a similar cavity of PTB in the right superior lobe. Outline along the outer edge of the cavity and include the cavity wall.

All images and clinical data were uploaded to Radiance's Radcloud platform (Huiying Medical Technology Co., Ltd) for further study. Two radiologists (reader 1: Q.Y, six years of experience, and reader 2: S.Q, ten years of experience) with expertise in chest disease were invited to interpret and delineate the cavities in the lung window CT images (W 1500, L -500). The two readers were both blinded to the clinical and pathogenic information. The first and last slices were excluded to eliminate the errors due to the volume effect, then reader 1 manually sketched the ROI by delineating the outline of the entire cavity on all contiguous slices. The contour line was drawn on the outer edge of the cavity and should completely include the cavity and its wall. If there were ground-glass opacities or consolidation partly next to the cavity, the outline was drawn along the existing outer wall based on its thickness and the shape of the inner wall. If there were multiple cavities in the patient's lungs, each cavity was individually outlined as an independent ROI, and if two or more lung cavities were connected or multilocular, they would be treated as one cavity and drawn at the outer wall, also cavities with diameters less than 0.5 cm were not drawn because of possible delineating error. A total of 379 ROIs were manually mapped out from the CT images of 182 patients. 142 patients were used for model training and internal validation, and 300 ROIS (164 NTM, 136 PTB) were labeled. 40 patients for external validation were labeled with 79 ROIS (49 NTM, 30 PTB). Subsequently, the second reader with a senior professional title then reviewed all the delineations. If the difference between the two readers was greater than or equal to 5%, the senior reader determined the cavity ROI boundary [18]. The contour lines of the cavity from the lung-window CT images are shown in Fig. 1a (NTM pulmonary disease) and Fig. 1b (PTB).

### Features extraction

A total of 1409 quantitative imaging features were extracted from ROIs with the RadCloud platform. These features were devided into three groups: Group 1 (intensity statistics features) consisted of 270 features that quantitatively delineated the distribution of voxel intensities within the CT images through commonly used and basic metrics. Group 2 (shape- and size-based features) contained 16 three-dimensional features that reflected the shape and size of the region. Group 3 (texture features) calculated by grey level run-length matrices (GLRLM), grey level co-occurrence texture matrices (GLCM), gray level size zone matrix (GLSZM), neighbouring gray tone difference matrix (NGTDM), and gray level dependence matrix (GLDM), and 1123 textural features that could quantify region heterogeneity differences were classified into group 3 (texture features).

### Features selection

As described above, high-throughput image features may be computed. However, not all the features were useful for a particular task. Therefore, in order to obtain the most relevant features, feature selection for the specific task was a necessary steps. Analysis of variance (ANOVA) and least absolute shrinkage and selection operator (LASSO) were used to remove redundant feature. ANOVA belongs univatiate feature selection method, using *P* value < 0.05 as the significant differences in features between the two lesions. For the LASSO method, L1 regularizer was used as the cost function, the error value of cross validation was 5, and the maximum number of iterations was 1000.

### Statistical analysis

The statistical analysis was performed in the Radcloud platform. After feature selection, a total of 66 features identified were significantly correlated to differentiating NTM pulmonary disease from PTB. Based on the selected features, there were several supervised learning classifiers available for classification analysis. In this study, the radiomics-based models were constructed with 6 classifiers, k-NearestNeighbor (KNN), Support Vector Machin (SVM), eXtreme Gradient Boosting (XGBoost), Random Forest (RF), Logistic Regression (LR) and Decision tree(DT), and the validation set was used to evaluate the effectiveness of these models. To assess the predictive performance, the receiver operating characteristic (ROC) curve and the area under curve (AUC) were used both in training set and validation set respectively. And four indicators including P (precision = true positives / (true positives + false positives)), R (recall = true positives / (true positives + false negatives)), f1-score (f1-score = P*R*2/ (P + R)), support (total number in test set) to evaluate the performance of classifier in this study.

## Results

**Fig. 2:**
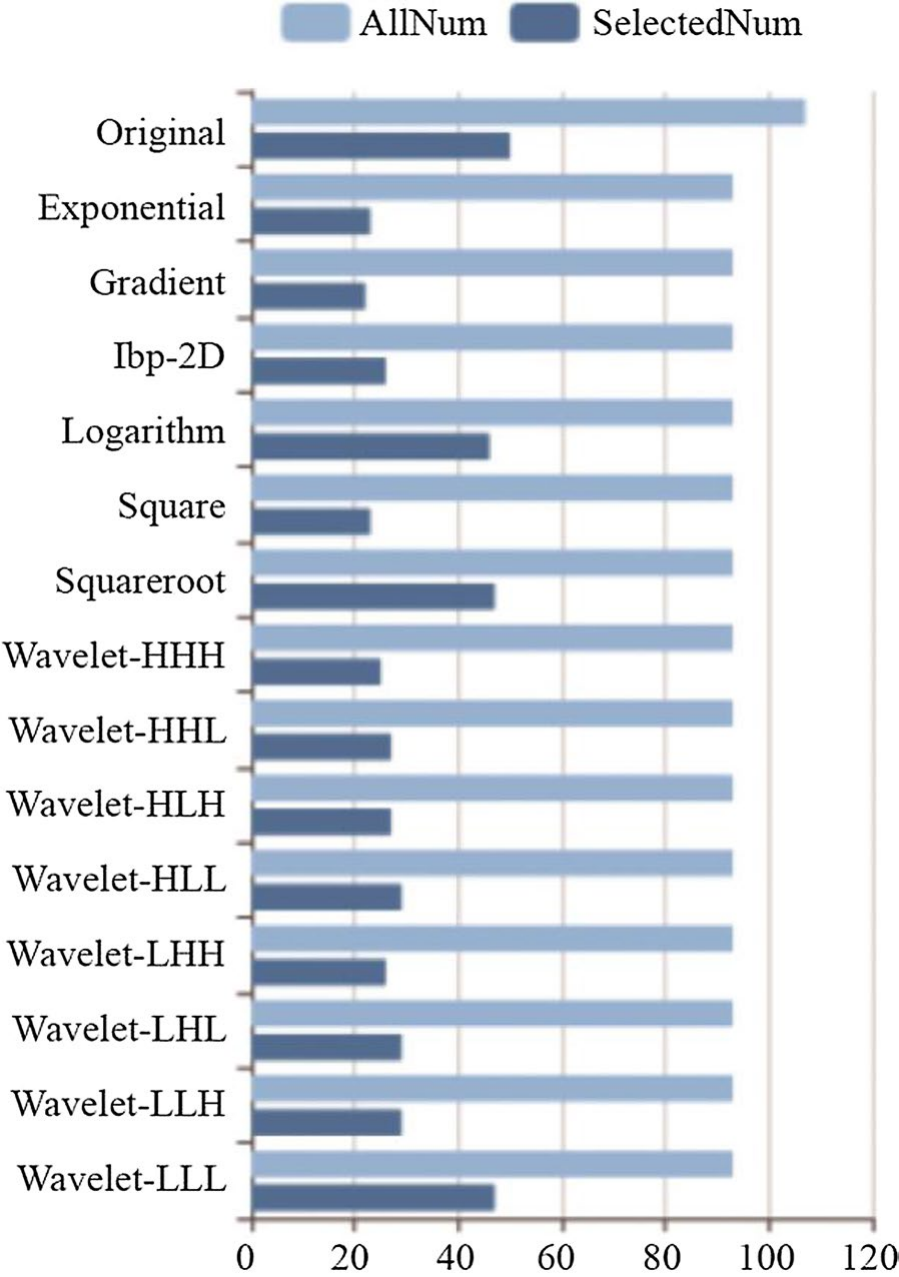
Variance threshold on feature select. We selected 857 radiomics features using variance threshold method (variance threshold = 0.8). The abscissa was the number of features, and the ordinate was the filtering method. Light blue in the bar indicates the number of original features, and dark blue indicated the number of filtered features

**Fig. 3:**
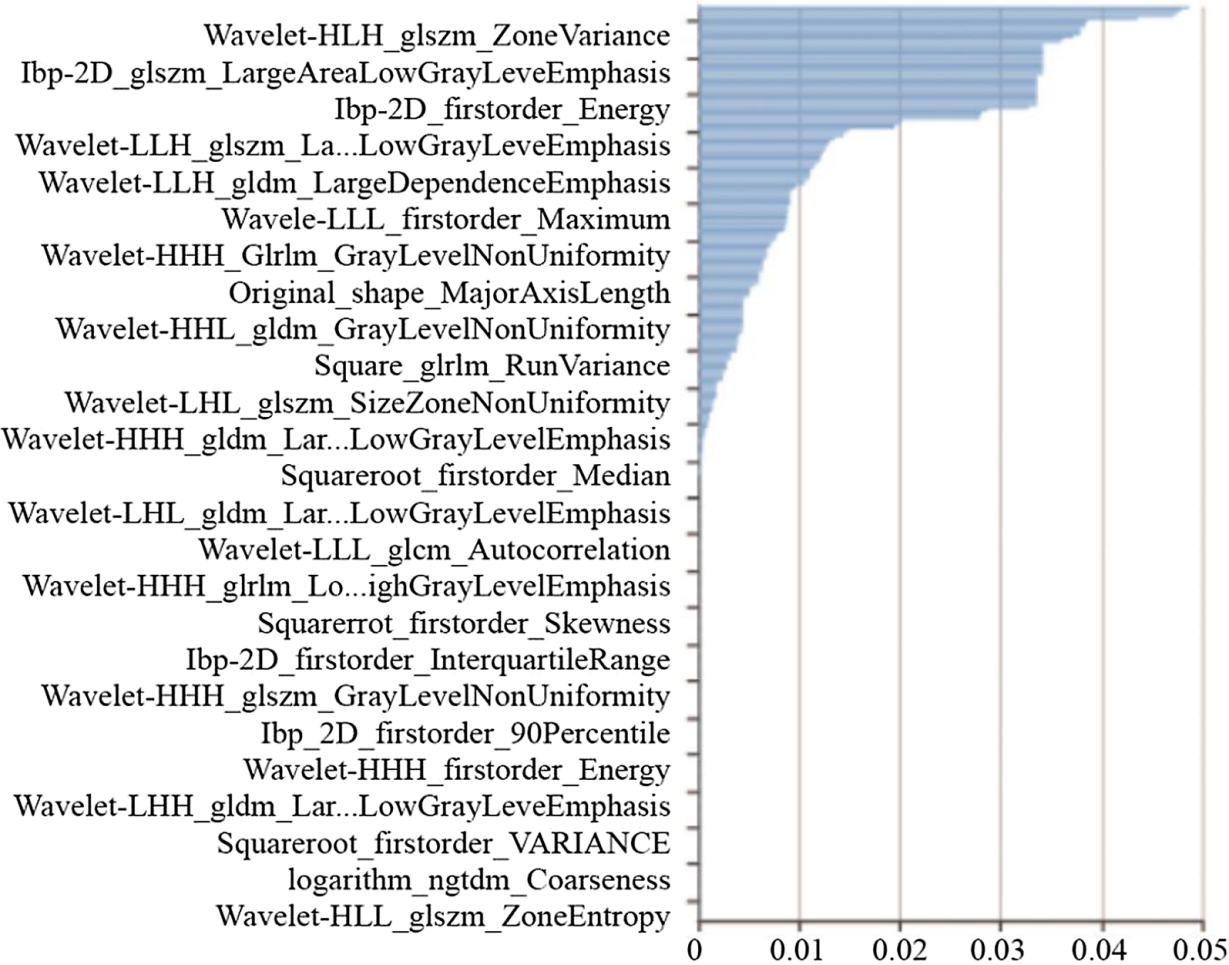
ANOVA on feature selectting. We used ANOVA method to further select radiomics features, and 631 features were selected. The abscissa was the *P*-value of the feature

**Fig. 4:**
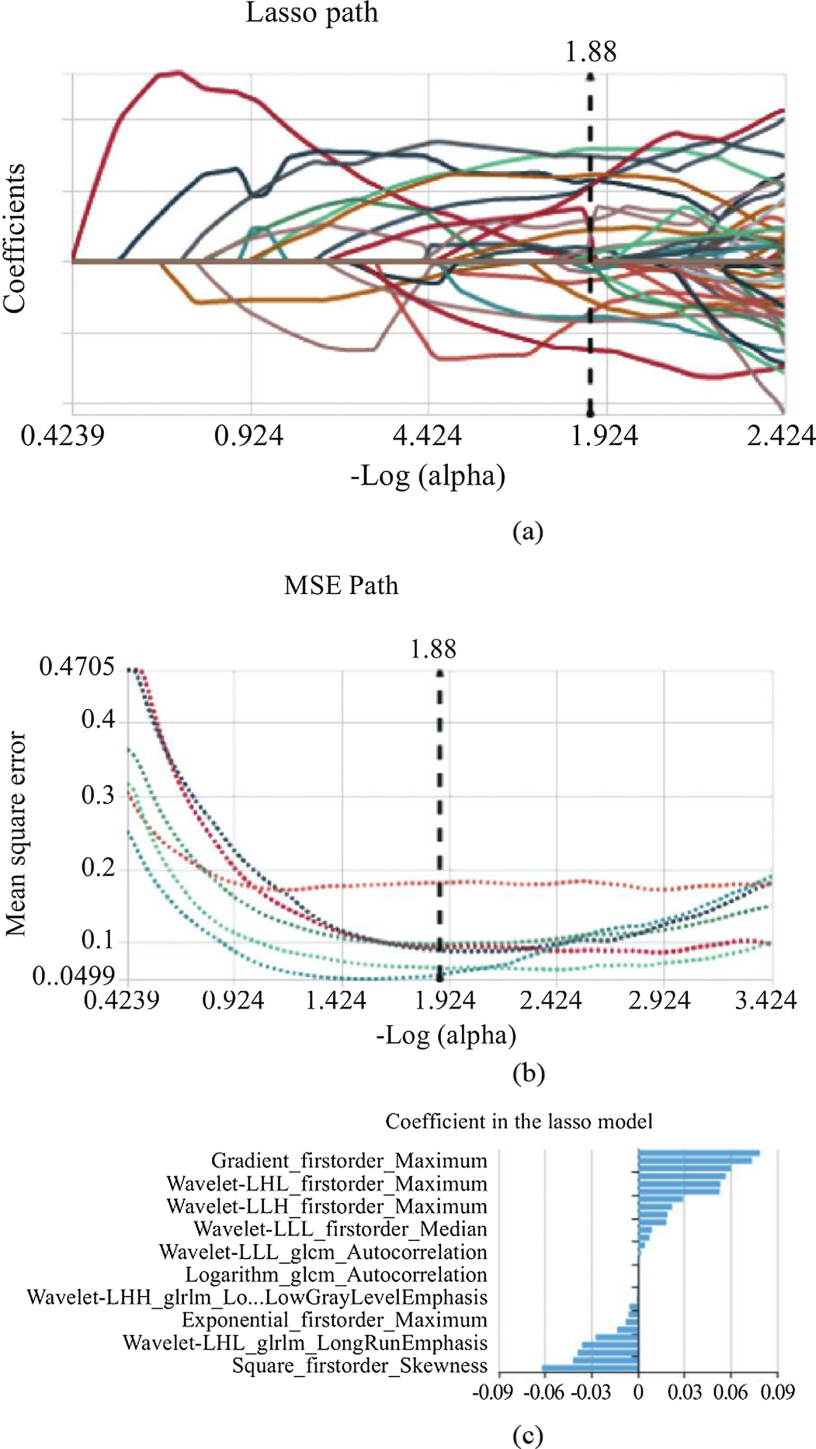
Lasso althorithm on feature selectting. **a** Laso path; **b** MSE path; **c** coefficients in Lass model. Using Lasso model, 66 features which were correspond to the optimal alpha value were selected

**Fig. 5:**
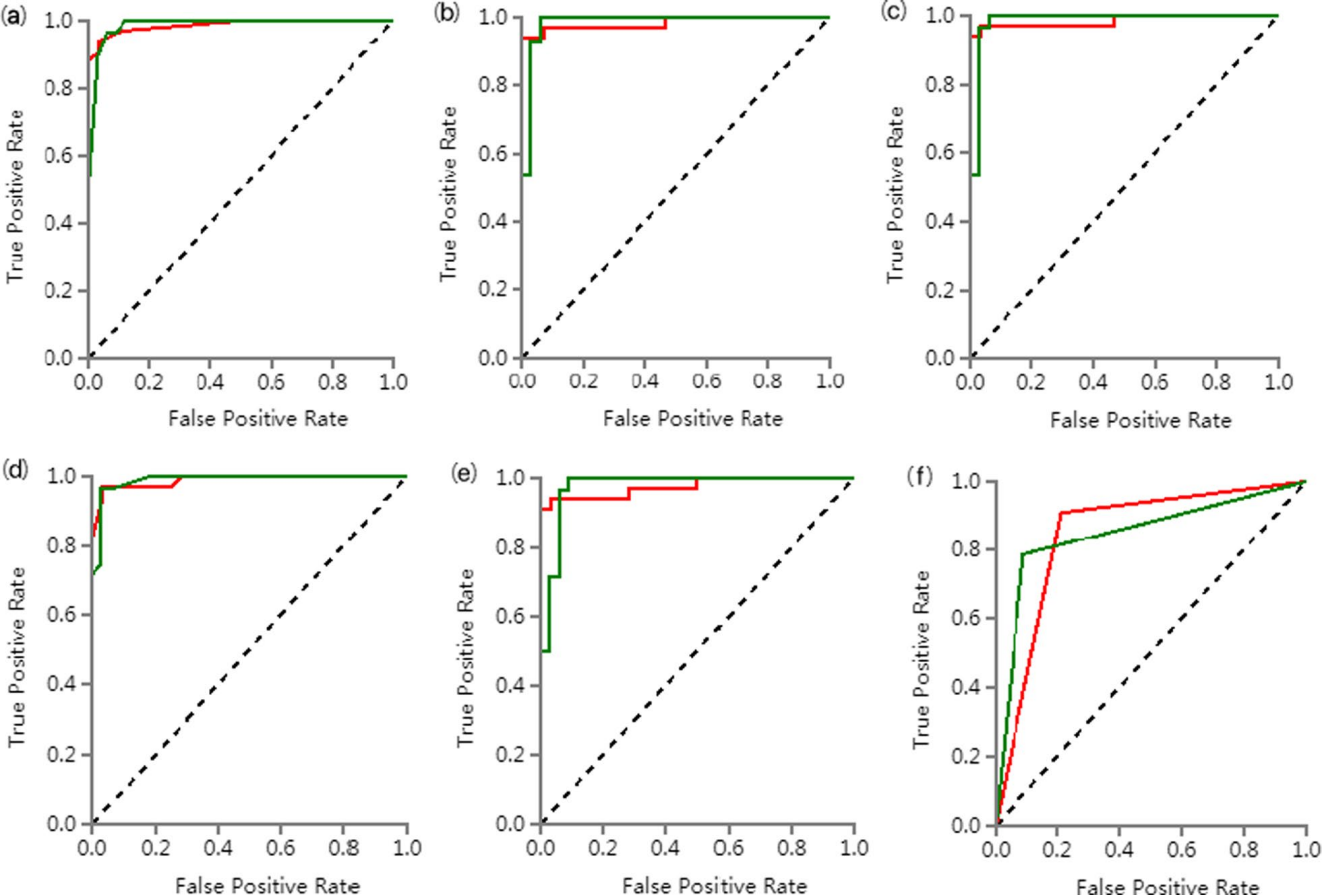
ROC curve of training set. **a** ROC curves of KNN methods to classification. **b** ROC curves of SVM methods to classification. **c** ROC curves of XGBoost methods to classification. **d** ROC curves of RF methods to classification. **e** ROC curves of LR methods to classification. **f** ROC curves of DT methods to classification

**Fig. 6:**
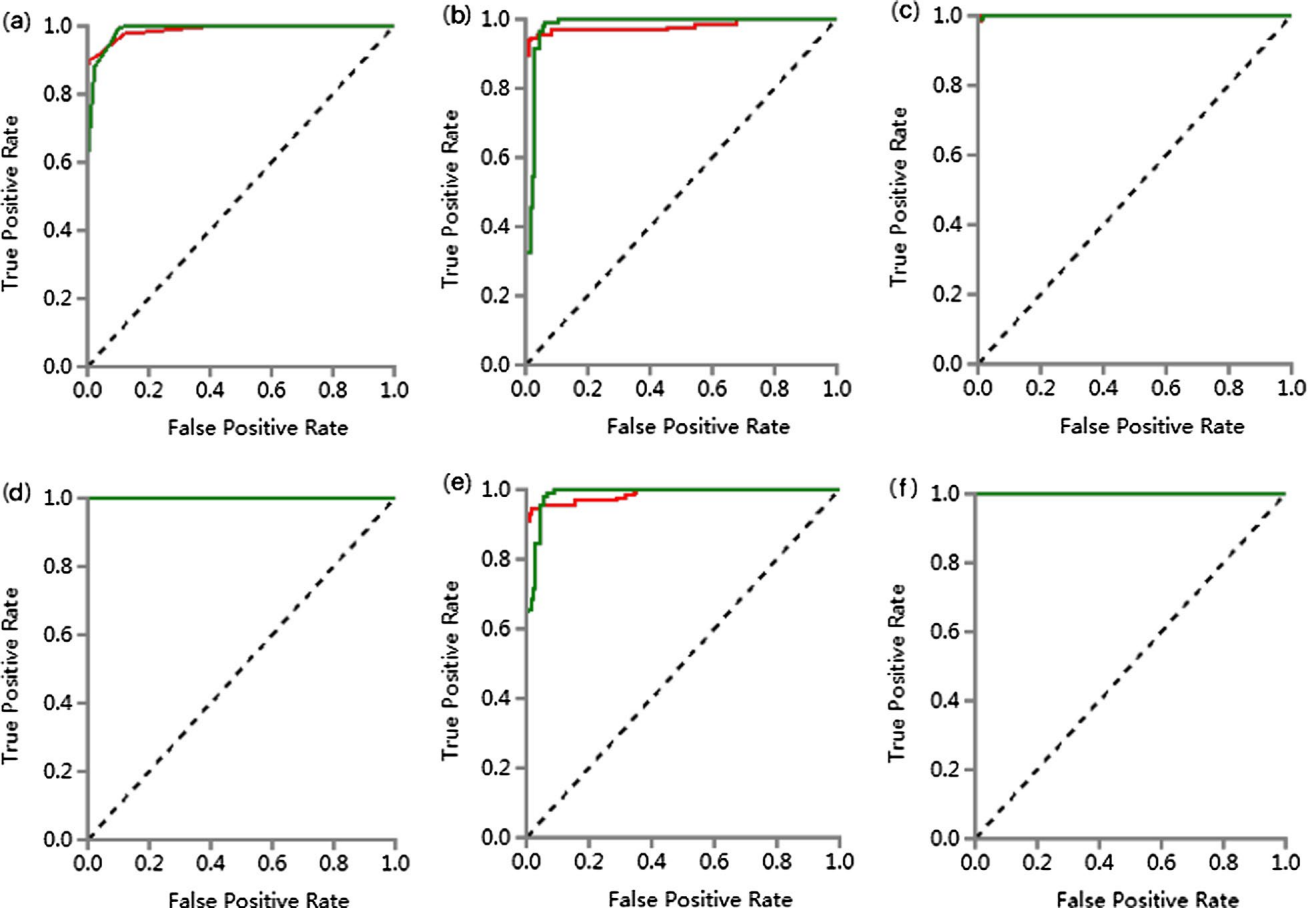
ROC curve of validation set. **a** ROC curves of KNN methods to classification; **b** ROC curves of SVM methods to classification; **c** ROC curves of XGBoost methods to classification; **d** ROC curves of RF methods to classification; **e** ROC curves of LR methods to classification; **f** ROC curves of DT methods to classification

Firstly, 476 features were selected from 1409 features using the variance threshold method (Fig. 2). Then 349 features were selected with the ANOVA method (Fig. 3). Finally, we selected 29 optimal features with LASSO algorithm (Fig. 4).When training with KNN SVM, XGBoost, RF, LR and DT classifier, the AUC of training set and validation set were shown in Figs. 5 and 6.

Six classifiers were used to analyze the characteristics of the radiomic AUC, the 95% CI, sensitivity, and specificity of the training and verification sets are shown in Tables 1 and 2. The AUC values of the six models in the training set were all greater than 0.98, with a 95% CI ranging between 0.95 to 1.00, the sensitivity was greater than 0.92, with a specificity value greater than 0.92. In the validation set, the AUC values of the six models were all greater than 0.85, with a 95% CI of 0.76–1.00, the sensitivity was greater than 0.79, and the specificity was greater than 0.79 furthermore, the results showed that irrespective of either the training set or the truth verification set, the AUC value was significantly high and greater than 0.85 in both instances. The AUC of the DT classifier was the lowest with a 95% CI of 0.76–0.94, sensitivity and specificity were greater than 0.79.

**Table 1:** ROC results with KNN, SVM, XGBoost, RF, LR and DT classifiers of training set

Classifiers	Category	AUC	95% CI	Sensitivity	Specificity
KNN	NTM	0.99	0.96–1.00	0.92	0.96
	TB	0.99	0.96–1.00	0.96	0.92
SVM	NTM	0.98	0.95–1.00	0.95	0.96
	TB	0.98	0.95–1.00	0.96	0.95
XGBoost	NTM	1.00	0.99–1.00	0.98	0.99
	TB	1.00	0.99–1.00	0.99	0.98
RF	NTM	1.00	0.99–1.00	0.99	1.00
	TB	1.00	0.99–1.00	1.00	0.99
LR	NTM	0.99	0.96–1.00	0.95	0.95
	TB	0.99	0.96–1.00	0.95	0.95
DT	NTM	1.00	1.00–1.00	1.00	1.00
	TB	1.00	1.00–1.00	1.00	1.00

t

**Table 2:** ROC results with six classifiers of validation set

Classifiers	Category	AUC	95% CI	Sensitivity	Specificity
KNN	NTM	0.99	0.93–1.00	0.94	0.96
	TB	0.99	0.93–1.00	0.96	0.94
SVM	NTM	0.98	0.91–1.00	0.97	0.82
	TB	0.98	0.91–1.00	0.82	0.97
XGBoost	NTM	0.98	0.94–1.00	0.97	0.96
	TB	0.98	0.94–1.00	0.96	0.97
RF	NTM	0.99	0.93–1.00	0.94	0.96
	TB	0.99	0.93–1.00	0.96	0.94
LR	NTM	0.98	0.91–1.00	0.94	0.93
	TB	0.98	0.91–1.00	0.93	0.94
DT	NTM	0.85	0.76–0.94	0.91	0.79
	TB	0.85	0.76–0.94	0.79	0.91

In the training set, the precision of the six models was greater than 0.90, the recall rate was greater than 0.92, the F1 score was greater than 0.93, and the support was 108 and 131(Table 3). In the validation set, the precision of the six models was greater than 0.83 with a recall rate greater than 0.79, the F1 score was greater than 0.83, and the support was 28 and 33(Table 4).

**Table 3:** The results of four indicators—precision, recall, F1-score, support in training set

	Indicators	KNN	SVM	XGBoost	RF	LR	DT
TB	Precision	0.90	0.95	0.98	0.99	0.94	1.00
	Recall	0.96	0.96	0.99	1.00	0.95	1.00
	F1-score	0.93	0.95	0.99	1.00	0.95	1.00
	Support	108	108	108	108	108	108
NTM	Precision	0.97	0.97	0.99	1.00	0.96	1.00
	Recall	0.92	0.95	0.98	0.99	0.95	1.00
	F1-score	0.94	0.96	0.99	1.00	0.96	1.00
	Support	131	131	131	131	131	131

**Table 4:** The results of four indicators—precision, recall, F1-score, support in validation set

	Indicators	KNN	SVM	XGBoost	RF	LR	DT
TB	Precision	0.93	0.96	0.96	0.93	0.93	0.88
	Recall	0.96	0.82	0.96	0.96	0.93	0.79
	F1-score	0.95	0.88	0.96	0.95	0.93	0.83
	Support	28	28	28	28	28	28
NTM	Precision	0.97	0.86	0.97	0.97	0.94	0.83
	Recall	0.94	0.97	0.97	0.94	0.94	0.91
	F1-score	0.95	0.91	0.97	0.95	0.94	0.87
	Support	33	33	33	33	33	33

Six classifiers were same used to analyze the characteristics of radiomic AUC, 95% CI, sensitivity and specificity of external validation set were shown in Table 5. The AUC values of the six models were all greater than 0.84, 95% CI were 0.64–1.00, the sensitivity was greater than 0.61, and the specificity was greater than 0.61. It was found from the table data that external validation set, The AUC value was similar to the results of internal validation, but the sensitivity, specificity and 95% CI were lower than those of internal validation. Among the six classifiers, LR classifier has the highest sensitivity, specificity and 95% CI, which were 0.94, 0.87, 0.84–0.96.

**Table 5:** ROC results with six classifiers of external validation set

Classifiers	Category	AUC	95% CI	Sensitivity	Specificity
KNN	NTM	0.84	0.66–0.82	0.65	0.83
	TB	0.84	0.66–0.82	0.83	0.65
SVM	NTM	0.90	0.78–0.92	0.94	0.77
	TB	0.90	0.78–0.92	0.77	0.94
XGBoost	NTM	0.96	0.75–0.87	0.97	0.65
	TB	0.96	0.75–0.87	0.65	0.97
RF	NTM	0.84	0.64–0.80	0.61	0.83
	TB	0.84	0.64–0.80	0.83	0.61
LR	NTM	0.95	0.84–0.96	0.94	0.87
	TB	0.95	0.84–0.96	0.87	0.94
DT	NTM	0.98	0.78–0.89	0.71	0.97
	TB	0.98	0.78–0.89	0.97	0.71

In the external validation set, the precision of the six models was greater than 0.57, the recall rate was greater than 0.61, the F1 score was greater than 0.68, and the support was 30 and 49 (Table 6). In the six classifiers, the LR classifier has the highest precision, recall, and F1-score.

**Table 6:** The results of four indicators—precision, recall, F1-score, support in external validation set

	Indicators	KNN	SVM	XGBoost	RF	LR	DT
TB	Precision	0.60	0.88	0.63	0.57	0.90	0.67
	Recall	0.83	0.77	0.97	0.83	0.87	0.97
	F1-score	0.69	0.82	0.76	0.68	0.88	0.79
	Support	30	30	30	30	30	30
NTM	Precision	0.86	0.87	0.97	0.86	0.92	0.97
	Recall	0.65	0.94	0.65	0.61	0.94	0.71
	F1-score	0.74	0.90	0.78	0.71	0.93	0.82
	Support	49	49	49	49	49	49

## Discussion

NTM pulmonary disease refers to lung diseases caused by mycobacteria except for *mycobacterium tuberculosis* complex and Mycobacterium leprae. Generally, these bacteria exist mainly in water and soil mediums, with over 190 NTM types identified in previous studies [17, 19–21], Among which only a small number of bacteria are disease-causing agents. The incidence and prevalence of NTM pulmonary disease are increasing in some countries and regions [22–25], with some regions reporting higher cases of NTM than PTB [3, 19]. The identification of NTM and PTB pulmonary diseases requires complex pathogenic testing. And the simple and non-invasive chest CT imaging has a very important role in diagnosis and differential diagnosis. The CT of NTM pulmonary disease shows a variety of signs such as cavity, and bronchiectasis, which makes it highly indistinguishable from PTB [26–28]. Studies have shown that cavity is a more common imaging manifestation in NTM pulmonary disease [29, 30]. There are plenty of literature results on the comparison of cavity types in pulmonary disease, with no consensus [8–11, 31]. Some studies [32] had shown that the cavities in NTM pulmonary disease are closer to the pleura of the lungs than those of PTB. Other studies [8, 9] pointed out that NTM pulmonary disease has thinner-walled cavities than PTB, with some researchers also reporting no statistically significant differences in the cavity wall results for the two diseases [6, 31]. This shows the existence of significant discrepancies in the use of conventional CT to identify the cavity features, especially its practical value.

Recent years have seen rapid developments in radiomics which converts medical images into high-dimensional images, and obtains quantifiable data features through quantitative high-throughput analysis, and then performs data analysis to provide clinical support. Radiomics has been shown to play a very important role in the diagnosis and differential diagnosis of clinical pulmonary diseases, such as the screening of small pulmonary nodules, diagnosis of lung cancer, the guidance of the treatment of pulmonary disease, and prognosis prediction [32–34]. Also, it has been well applied in the diagnosis of some common infectious diseases [35, 36]. However, there are relatively few studies on diffuse pulmonary disease [35, 37], and we assume this is due to the difficulty in the selection and delineation of the target area. The CT imaging of NTM pulmonary disease is complex and variable, including cavity signs, bronchiectasis, bronchitis, pulmonary consolidation, etc., the lesions exist alone or at the same time. These CT features were similar to pulmonary tuberculosis and difficult to distinguish. Some studies showned that the signs of bronchiectasis and cavity in CT images of NTM pulmonary diseases were different from those of pulmonary tuberculosis [28–30]. In this study, 62 of the 73 patients with NTM pulmonary disease were complicated with bronchiectasis, 58 of the 69 patients with NTM pulmonary disease were complicated with bronchiectasis. Since cavities were easy to observe and outline. besides, studies have shown that cavity has significant associations with mortality [38, 39], disease progression, and the failure of clinical treatment [40, 41], hence, the indication by the international guidelines for the diagnosis and treatment of NTM pulmonary disease indicate that patients with cavity need active treatment [42]. Therefore, in this study, cavities were used as the target areas for radiomics analysis, and the radiomics features obtained from cavities were used to explore the significance of identifying the disease from PTB. Radiomics can extract valuable cavity radiomics information from CT images for the differential diagnosis of the two diseases, which can improve the early diagnostic rate of NTM pulmonary disease and prevent misdiagnosis and mistreatment. The Xpert and PCR laboratory tests are DNA tests of *Mycobacterium tuberculosis* used to diagnose tuberculosis infection, but cannot diagnose NTM infection. Radiomics can be used in conjunction with the laboratory tests such as Xpert and PCR to prevent early and efficient diagnosis and treatment. Early diagnosis of NTM pulmonary disease can improve the quality of the patient’s life and promote the treatment of diseases, especially for the resource deficient health care system in developing countries [43, 44].

In this study, the cavities of NTM pulmonary disease and PTB were used as potential areas of interest for delineation. A total of 29 radiomics features were obtained from ROIs, of which 14 were texture analysis and 15 were first-order statistical feature groups. The study used six supervised learning classifiers (KNN, SVM, XGBoost, RF, and LRandDT) to analyze the extracted lung cavity features. In the training set, the AUC values of the six models were all greater than 0.98, 95% CI were 0.95–1.00, the sensitivity was greater than 0.92, and the specificity was greater than 0.92. In the validation set, the AUC values of the six models were all greater than 0.85, 95% CI were 0.76–1.00, the sensitivity was greater than 0.79, and the specificity was greater than 0.79. The obtaidne AUC values of ROC curve were all high, and their sensitivity and specificity were greater than 0.79. In the external validation set, The AUC value was similar to the results of internal validation, but the sensitivity, specificity and 95% CI were lower than those of internal validation. Among the six classifiers, LR classifier has highest sensitivity and specificity, which were 0.94, 0.87. The study analyzed the characteristics of the six classifiers through four clinical indicators (accuracy, recall, f1 points, and support).In the training set, the precision of the six models was greater than 0.90, the recall rate was greater than 0.92, the F1 score was greater than 0.93, and the support were 108 and 131. In the validation set, the precision of the six models was greater than 0.83, the recall rate was greater than 0.79, the F1 score was greater than 0.83, and the support were 28 and 33. In the external validation set, the precision of the six models was greater than 0.57, the recall rate was greater than 0.61, the F1 score was greater than 0.68. Furthermore, the LR classifier has the highest precision, recall, and F1-score, which were 0.92, 0.94, 0.93 respectively in NTM group. The results showed that the radiomics features derived from the cavities could help in differentiating between the NTM pulmonary disease and PTB. Although some studies have reported that the CT imaging characteristics of NTM pulmonary disease cavities observed through traditional clinical methods differ from those of PTB [17, 45], the results obtained by researchers' naked eye recognition may appear subjective. Consequently, the effect is significantly reduced in actual clinical applications. The radiomics analysis of the cavity characteristics of pulmonary disease has the advantages of objectiveness, quantification, stability, and nonexperience dependence. Therefore, it presents a significant value for clinical application. It can be seen that the use of the radiomics characteristics in differentiating NTM pulmonary disease from PTB is very promising, and the results may help clinical radiologists lacking experience or have difficulty in visual recognition. Therefore, this technology is a very important, non-invasive, simple, and easy method in the identification of these two diseases.

In addition, although the pathological features of NTM pulmonary disease and tuberculosis are similar, there are some differences as follows; (1) the lesions of the NTM pulmonary disease have less caseous necrosis than PTB; (2) epithelial-like cells aggregation tends to be proliferative granuloma; and (3) the cavities are more common and the characteristics may be in multiple forms, multiple rooms and uneven inner walls [15]. Thus, the subtle differences in these pathological features of conventional CT images are difficult to be distinguished, resulting in a low level of differential diagnosis from PTB. The high-throughput extracted CT data of radiomics can reflect the internal differences of the pathological characteristics of the two diseases, which is consistent with the pathological performance and makes up for the lack of visual observation and key information in CT images.

However, this study also has some limitations: 1. to ensure the homogeneity of the images, images with 5 mm layer thickness were used in the research process. Compared with thinner slice images, some information may be lost. 2. ROIs were delineated manually and individual subjectivity is unavoidable. 3. We focused only on the cavity as the research target and ignored other image features, which could lead to incomplete information and the inability to study some typical patients. 4. Increase the amount of data and further improve the data analysis model. The above shortcomings and deficiencies will be the focus of future research.

Conclusively, this study shows that the radiomics features based on CT imaging are effective in identifying NTM pulmonary disease cavities and PTB cavities, which is highly significant for early non-invasive diagnosis and differentiation of NTM pulmonary disease with cavity. Among the six classifiers, the LR classifier has the best performance in identifying the two diseases.

## Supplementary Information

**Additional file 1.** Data model and results in this study.

**Additional file 2.** External validation of the model in this study.

**Additional file 3.** Ethical proof.

## Data Availability

The datasets used and/or analysed during the current study are available from the corresponding author on reasonable request.
