# Supplementary material for: Differentiating nontuberculous mycobacterium pulmonary disease from pulmonary tuberculosis through the analysis of the cavity features in CT images using radiomics
Source: BMC Pulm Med. 2022 Jan 7;22:4. doi: 10.1186/s12890-021-01766-2 (PMC8740493; doi:10.1186/s12890-021-01766-2)
Supplement: Supplementary file 1 — Additional file 1. Data model and results in this study. [file 12890_2021_1766_MOESM1_ESM.pdf]

Differentiating Nontuberculous Mycobacterium pulmonary Disease from Pulmonary Tuberculosis through the Analysis of the Cavity features in CT images using Radiomics

Radcloud Analysis Report

1. Introduction

Radiomics is an emerging field that aims at building a relevant statistical model from a large number of high-dimensional mineable features extracted from medical imaging data (possibly combined with clinical or genomic data) to assist diagnosis, prognosis and therapy monitoring.

Radiomics workflow involves: Imaging, ROI segmentation, feature extraction and analysis, then with the selected features, a statistical model is designed based on machine learning algorithms, which have to be tuned according to the clinical or biological question and to the a priori knowledge that is available.

2. Materials and Methods

2.1 Patients and Dataset

A total of 0 patients(114 men and 61 women; maen age, 51 years ±17.44; range, 17-90 years) were included in this study. We used Radcloud (Huiying Medical Technology Co., Ltd) to manage imaging data, clinical data, and subsequent radiomics statistics analysis.

The training dataset and validation dataset were separated by random method with ratio 2:8, and the random seeds is 687

2.2 Image segmentation

2.3 Feature extraction

A total of 1409 quantitative imaging features were extracted from CT images with Radcloud platform (http://radcloud.cn/). These features can be grouped into three groups. Group 1 (first order statistics) consisted of 126 descriptors that quantitatively delineate the distribution of voxel intensities within the CT image through commonly used and basic metrics. Group 2 (shape- and size-based features) contained 14 three-dimensional features that reflect the shape and size of the region. Calculated from grey level run-length and grey level co-occurrence texture matrices, 525 textural features that can quantify region heterogeneity differences were classified into group 3 (texture features).

2.4 Feature qualification

As described above, a large number of image features may be computed. However, all these extracted features may not be useful for a particular task. Therefore, dimensionality reduction and selection of task-specific features for best performance are necessary steps. To reduce the redundant features, the feature selection methods included the variance threshold (variance threshold = 0.8), SelectKBest and the least absolute shrinkage and selection operator (LASSO) were used for this purpose. For the variance threshold method, the threshold is 0.8, so that the eigenvalues of the variance smaller than 0.8 were removed. The SelectKBest method, which belongs to a single variable feature selection method, using p value to analysis the relationship between the features and the classification results, all the features with p value smaller than 0.05 will be used. For LASSO model, L1 regularizer was used as the cost function, and the error value of cross validation is 5, and the maximum number of iterations is 1000.

2.5 Statistical analysis

The statistical analysis was performed in Radcloud platform. And after feature qualification, a total of 1409 features identified were significantly correlated to this subject. Based on the selected features, there are several supervised learning classifiers available for classification analysis, which creates models that attempt to separate or predict the data with respect to an outcome or phenotype (for instance, patient outcome or response). In this study, the radiomics-based models were constructed with 6 classifiers, k-NearestNeighbor(KNN), Support Vector Machin(SVM), eXtreme Gradient Boosting(XGBoost), Random Forest (RF), Logistic Regression (LR) and Decision tree(DT), and the validation method was used to improve the effectiveness of the model.

For KNN, the parameters KNN paramsters: n_neighbors(5), weights(uniform),

For SVM, the parameters SVM paramsters: kernel(rbf), C(1), gamma(auto), class_weight(balanced), decision_function_shape(ovr), random_state(),

For XGBoost, the parameters XGBoost paramsters: Eta(0.3), max_depth(6),

For RF, the parameters RF paramsters: n_estimators(10), class_weight(None),

For LR, the parameters LR paramsters: penalty(L2), C(1), solver(liblinear), class_weight(None), multi_class(ovr), random_state(),

For DT, the parameters DT paramsters: splitter(best), criterion(gini),

To assess the predictive performance, the receiver operating characteristic (ROC) curve, namely, area under curve (AUC) were used both in training dataset and validation dataset respectively. And four indicators including P (precision = true positives / (true positives+ false positives)), R (recall = true positives / (true positives+ false negatives)), f1-score (f1-score = P*R*2/ (P+R)), support (total number in test set) to evaluate the performance of classifier in this study.

3.Results

We firstly select 476 features from 1409 features using variance threshold method (Fig. 1), then with the select K best methods, we select 349 features (Fig. 2), finally, we selected 29 optimal features (Table -1) with LASSO algorithm (Fig. 3).

ROC curve analysis results were showed in Tab. 2 for training set and Tab. 3 for validation set. When training with KNN classifier, the AUC of training set were 0.988690981057 in NTM (95% CI: 0.96 - 1.00; sensitivity 0.92 and specificity 0.96), 0.988690981057 in TB (95% CI: 0.96 - 1.00; sensitivity 0.96 and specificity 0.92), respectively, the AUC of validation set were 0.987012987013 in NTM (95% CI: 0.93 - 1.00; sensitivity 0.94 and specificity 0.96), 0.987012987013 in TB (95% CI: 0.93 - 1.00; sensitivity 0.96 and specificity 0.94), respectively (Figure 4).training with SVM classifier, the AUC of training set were 0.979926491377 in NTM (95% CI: 0.95 - 1.00; sensitivity 0.95 and specificity 0.96), 0.979926491377 in TB (95% CI: 0.95 - 1.00; sensitivity 0.96 and specificity 0.95), respectively, the AUC of validation set were 0.983766233766 in NTM (95% CI: 0.91 - 1.00; sensitivity 0.97 and specificity 0.82), 0.983766233766 in TB (95% CI: 0.91 - 1.00; sensitivity 0.82 and specificity 0.97), respectively (Figure 5).training with XGBoost classifier, the AUC of training set were 0.999858637263 in NTM (95% CI: 0.99 - 1.00; sensitivity 0.98 and specificity 0.99), 0.999858637263 in TB (95% CI: 0.99 - 1.00; sensitivity 0.99 and specificity 0.98), respectively, the AUC of validation set were 0.984848484848 in NTM (95% CI: 0.94 - 1.00; sensitivity 0.97 and specificity 0.96), 0.984848484848 in TB (95% CI: 0.94 - 1.00; sensitivity 0.96 and specificity 0.97), respectively (Figure 6).training with RF classifier, the AUC of training set were 0.999964659316 in NTM (95% CI: 0.99 - 1.00; sensitivity 0.99 and specificity 1.0), 0.999964659316 in TB (95% CI: 0.99 - 1.00; sensitivity 1.00 and specificity 0.99), respectively, the AUC of validation set were 0.988636363636 in NTM (95% CI: 0.93 - 1.00; sensitivity 0.94 and specificity 0.96), 0.988636363636 in TB (95% CI: 0.93 - 1.00; sensitivity 0.96 and specificity 0.94), respectively (Figure 7).training with LR classifier, the AUC of training set were 0.986853265479 in NTM (95% CI: 0.96 - 1.00; sensitivity 0.95 and specificity 0.95), 0.986853265479 in TB (95% CI: 0.96 - 1.00; sensitivity 0.95 and specificity 0.95), respectively, the AUC of validation set were 0.975108225108 in NTM (95% CI: 0.91 - 1.00; sensitivity 0.94 and specificity 0.93), 0.975108225108 in TB (95% CI: 0.91 - 1.00; sensitivity 0.93 and specificity 0.94), respectively (Figure 8).training with DT classifier, the AUC of training set were 1.0 in NTM (95% CI: 1.00 - 1.00; sensitivity 1.00 and specificity 1.0), 1.0 in TB (95% CI: 1.00 - 1.00; sensitivity 1.00 and specificity 1.0), respectively, the AUC of validation set were 0.847402597403 in NTM (95% CI: 0.76 - 0.94; sensitivity 0.91 and specificity 0.79), 0.847402597403 in TB (95% CI: 0.76 - 0.94; sensitivity 0.79 and specificity 0.91), respectively (Figure 9).

We summarized these four indicators (precision, recall, f1-score, support) for classifiers in Tab. 4 and Tab. 5, respectively. When training training with KNN classifier, the precision, recall, f1-score and support of training set were 0.97, 0.92, 0.94 and 131 in NTM, 0.90, 0.96, 0.93 and 108 in TB, the precision, recall, f1-score and support of validation set were 0.97, 0.94, 0.95 and 33 in NTM and 0.93, 0.96, 0.95 and 28 in TB. training with SVM classifier, the precision, recall, f1-score and support of training set were 0.97, 0.95, 0.96 and 131 in NTM, 0.95, 0.96, 0.95 and 108 in TB, the precision, recall, f1-score and support of validation set were 0.86, 0.97,

0.91 and 33 in NTM and 0.96, 0.82, 0.88 and 28 in TB. training with XGBoost classifier, the precision, recall, f1-score and support of training set were 0.99, 0.98, 0.99 and 131 in NTM, 0.98, 0.99, 0.99 and 108 in TB, the precision, recall, f1-score and support of validation set were 0.97, 0.97, 0.97 and 33 in NTM and 0.96, 0.96, 0.96 and 28 in TB. training with RF classifier, the precision, recall, f1-score and support of training set were 1.00, 0.99, 1.00 and 131 in NTM, 0.99, 1.00, 1.00 and 108 in TB, the precision, recall, f1-score and support of validation set were 0.97, 0.94, 0.95 and 33 in NTM and 0.93, 0.96, 0.95 and 28 in TB. training with LR classifier, the precision, recall, f1-score and support of training set were 0.96, 0.95, 0.96 and 131 in NTM, 0.94, 0.95, 0.95 and 108 in TB, the precision, recall, f1-score and support of validation set were 0.94, 0.94, 0.94 and 33 in NTM and 0.93, 0.93, 0.93 and 28 in TB. training with DT classifier, the precision, recall, f1-score and support of training set were 1.00, 1.00, 1.00 and 131 in NTM, 1.00, 1.00, 1.00 and 108 in TB, the precision, recall, f1-score and support of validation set were 0.83, 0.91, 0.87 and 33 in NTM and 0.88, 0.79, 0.83 and 28 in TB.

Tab. 1 Description of the selected radiomic features with their associated feature group and filter

Radiomic feature	Radiomic class	Filter
RobustMeanAbsoluteDeviation	firstorder	lbp-2D
Variance	firstorder	lbp-2D
Kurtosis	firstorder	lbp-2D
90Percentile	firstorder	lbp-2D
LongRunLowGrayLevelEmphasis	glrlm	wavelet-LHH
Maximum	firstorder	gradient
Maximum	firstorder	wavelet-LHL
LongRunHighGrayLevelEmphasis	glrlm	wavelet-HLH
Range	firstorder	wavelet-HLL
LongRunEmphasis	glrlm	wavelet-LHL
LongRunLowGrayLevelEmphasis	glrlm	wavelet-HLL
LongRunLowGrayLevelEmphasis	glrlm	wavelet-LHL
Autocorrelation	glcm	wavelet-LLL

SumAverage	glcm	original
SumAverage	glcm	logarithm
Autocorrelation	glcm	original
Autocorrelation	glcm	logarithm
Autocorrelation	glcm	squareroot
Range	firstorder	wavelet-HLH
Median	firstorder	original
Median	firstorder	wavelet-LLL
ZoneEntropy	glszm	wavelet-HHL
Maximum	firstorder	wavelet-LLH
Skewness	firstorder	square
Energy	firstorder	wavelet-HHL
HighGrayLevelZoneEmphasis	glszm	wavelet-LLL
Maximum	firstorder	exponential
Maximum	firstorder	wavelet-LLL
LongRunEmphasis	glrlm	wavelet-LLH

Label: GLCM=Gray-level Co-occurrence Matrix,GLRLM=Gray Level Run Length Matrix,GLSZM=Gray-Level Size Zone Matrix

Tab. 2 ROC results with KNN, SVM, XGBoost, RF, LR and DT classifiers of training set

Classifiers	Category	AUC	95% CI	Sensitivity	Specific
KNN	NTM	0.9886909810573932	0.96 - 1.00	0.92	0.96
	TB	0.9886909810573933	0.96 -	0.96	0.92

			1.00		
SVM	NTM	0.979926491376873	0.95 - 1.00	0.95	0.96
	TB	0.9799264913768732	0.95 - 1.00	0.96	0.95
XGBoost	NTM	0.9998586372632173	0.99 - 1.00	0.98	0.99
	TB	0.9998586372632174	0.99 - 1.00	0.99	0.98
RF	NTM	0.9999646593158045	0.99 - 1.00	0.99	1
	TB	0.9999646593158044	0.99 - 1.00	1.00	0.99
LR	NTM	0.9868532654792197	0.96 - 1.00	0.95	0.95
	TB	0.9868532654792197	0.96 - 1.00	0.95	0.95
DT	NTM	1	1.00 - 1.00	1.00	1
	TB	1	1.00 - 1.00	1.00	1

Tab. 3 ROC results with six classifiers of validation set

Classifiers	Category	AUC	95% CI	Sensitivity	Specific
KNN	NTM	0.987012987012987	0.93 - 1.00	0.94	0.96
	TB	0.987012987012987	0.93 - 1.00	0.96	0.94
SVM	NTM	0.9837662337662338	0.91 - 1.00	0.97	0.82
	TB	0.9837662337662338	0.91 -	0.82	0.97

			1.00		
XGBoost	NTM	0.9848484848484849	0.94 - 1.00	0.97	0.96
	TB	0.9848484848484849	0.94 - 1.00	0.96	0.97
RF	NTM	0.9886363636363635	0.93 - 1.00	0.94	0.96
	TB	0.9886363636363636	0.93 - 1.00	0.96	0.94
LR	NTM	0.9751082251082253	0.91 - 1.00	0.94	0.93
	TB	0.9751082251082251	0.91 - 1.00	0.93	0.94
DT	NTM	0.8474025974025974	0.76 - 0.94	0.91	0.79
	TB	0.8474025974025973	0.76 - 0.94	0.79	0.91

Tab. 4 The results of four indicators -Precision, Recall, F1-score, Support in training set

	**Indicators**	**KNN**	**SVM**	**XGBoost**	**RF**	**LR**	**DT**
TB	Precision	0.90	0.95	0.98	0.99	0.94	1.00
	Recall	0.96	0.96	0.99	1.00	0.95	1.00
	F1-score	0.93	0.95	0.99	1.00	0.95	1.00
	Support	108	108	108	108	108	108
NTM	Precision	0.97	0.97	0.99	1.00	0.96	1.00
	Recall	0.92	0.95	0.98	0.99	0.95	1.00
	F1-score	0.94	0.96	0.99	1.00	0.96	1.00

	Support	131	131	131	131	131	131

Tab. 5 The results of four indicators -Precision, Recall, F1-score, Support in validation set

	**Indicators**	**KNN**	**SVM**	**XGBoost**	**RF**	**LR**	**DT**
TB	Precision	0.93	0.96	0.96	0.93	0.93	0.88
	Recall	0.96	0.82	0.96	0.96	0.93	0.79
	F1-score	0.95	0.88	0.96	0.95	0.93	0.83
	Support	28	28	28	28	28	28
NTM	Precision	0.97	0.86	0.97	0.97	0.94	0.83
	Recall	0.94	0.97	0.97	0.94	0.94	0.91
	F1-score	0.95	0.91	0.97	0.95	0.94	0.87
	Support	33	33	33	33	33	33

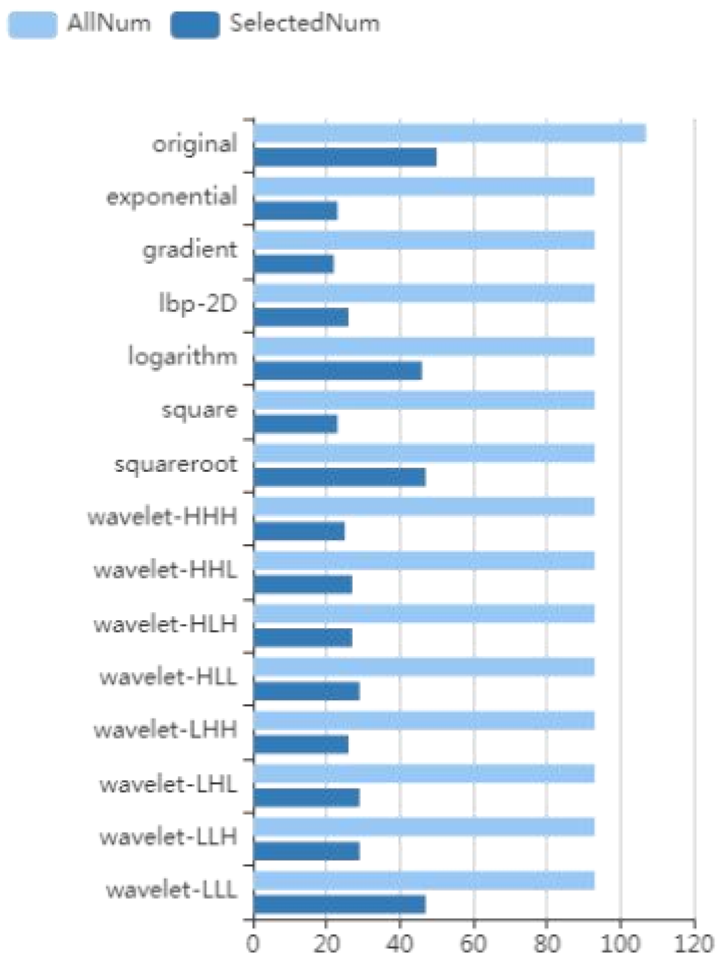

Fig. 1. Variance threshold on feature select. We used variance threshold methods to select radiomics features (variance threshold = 0.8), we selected 476 features from 1409 features.

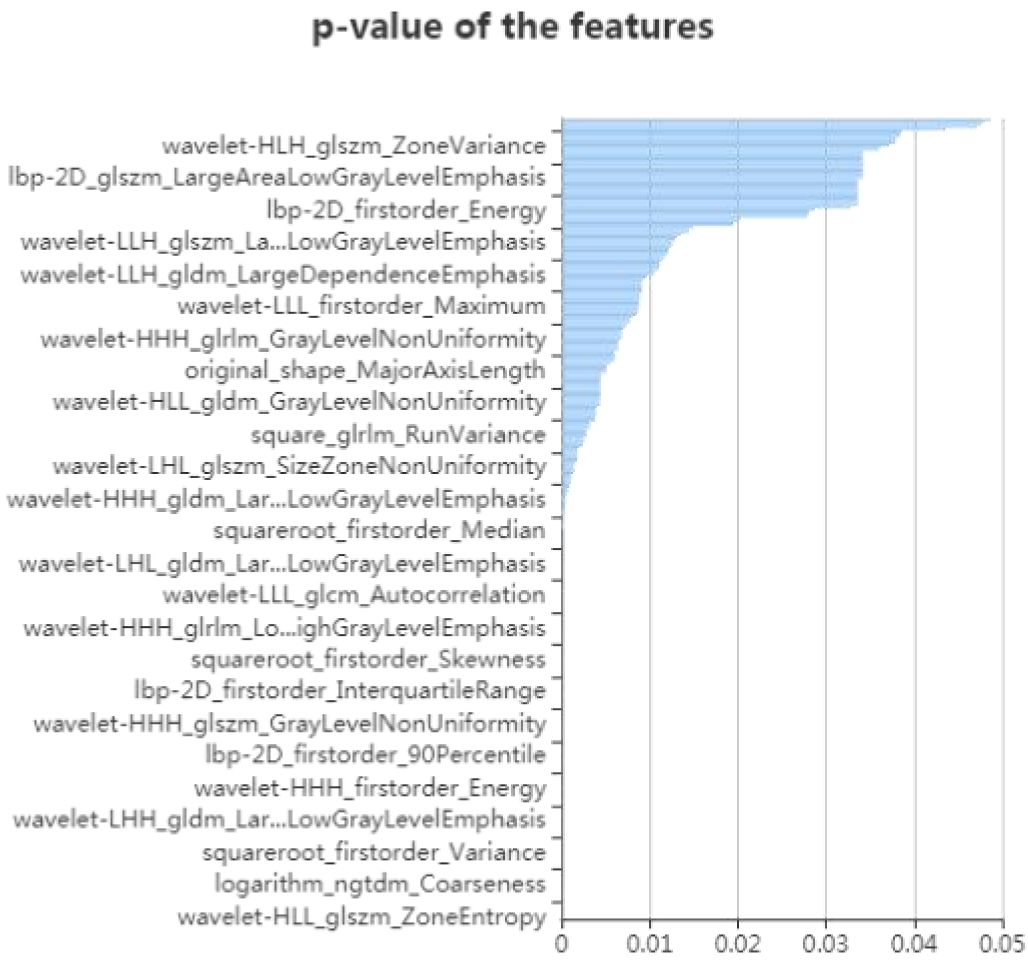

Fig. 2. Select K best on feature select. We used Select K best methods to further select radiomics features, we selected 349 features.

Lasso parameters: cv(5) and max_iter(1000)

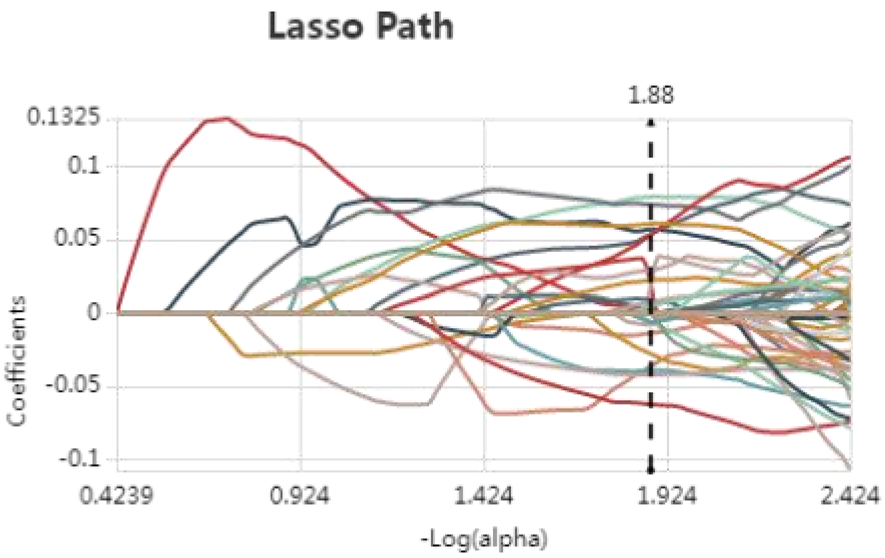
Lasso Path
1.88
0.1325
0.1
0.05
0
-0.05
-0.1
Coefficients
0.4239
0.924
1.424
1.924
2.424
-Log(alpha)

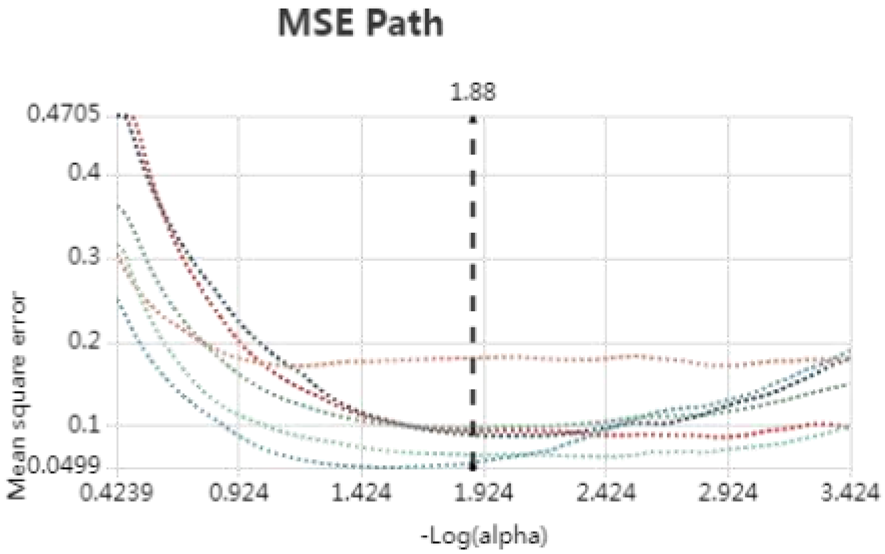
MSE Path
1.88
0.4705
0.4
0.3
0.2
0.1
0.0499
Mean square error
0.4239
0.924
1.424
1.924
2.424
2.924
3.424
-Log(alpha)

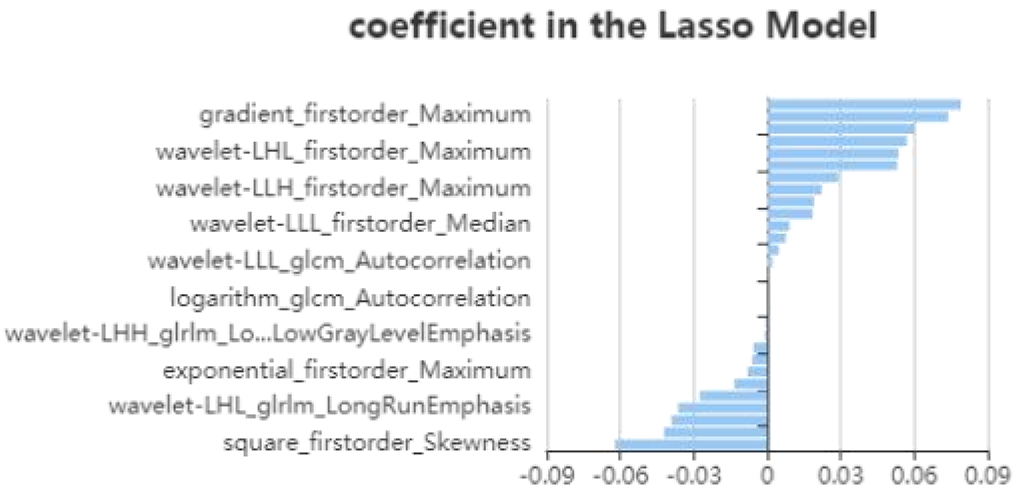
coefficient in the Lasso Model
gradient_firstorder_Maximum
wavelet-LHL_firstorder_Maximum
wavelet-LLH_firstorder_Maximum
wavelet-LLL_firstorder_Median
wavelet-LLL_glcm_Autocorrelation
logarithm_glcm_Autocorrelation
wavelet-LHH_glrlm_Lo...LowGrayLevelEmphasis
exponential_firstorder_Maximum
wavelet-LHL_glrlm_LongRunEmphasis
square_firstorder_Skewness
-0.09
-0.06
-0.03
0
0.03
0.06
0.09

Fig. 3. Lasso althorithm on feature select. (a) Laso path; (b) MSE path; (c) coefficients in Lass model. Using Lasso model, 29 features which are correspond to the optimal alpha value were selected.

KNN paramsters: n_neighbors(5), weights(uniform),

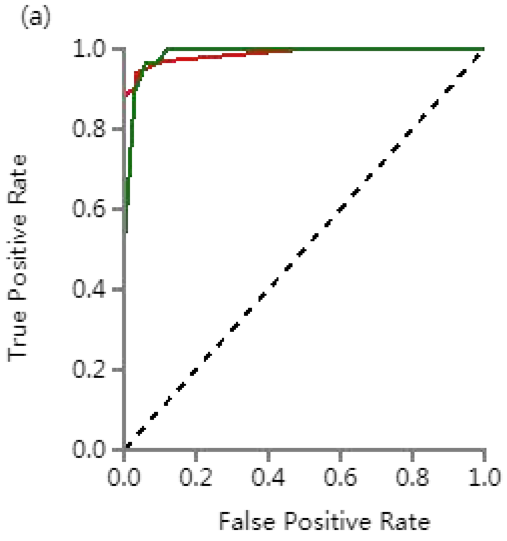

NTM(auc=0. 99) TB(auc=0. 99)

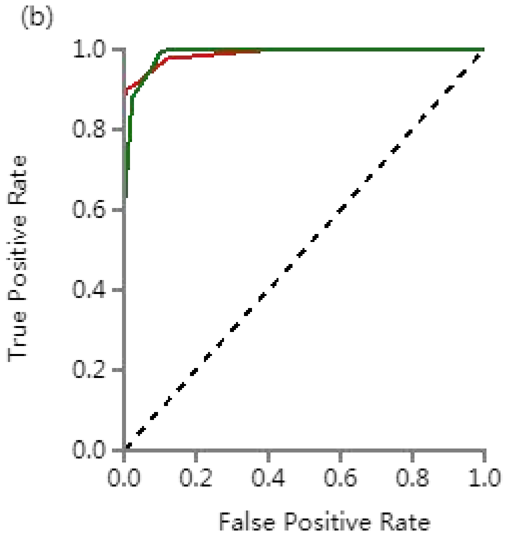

NTM(auc=0. 99) TB(auc=0. 99)

Fig. 4. ROC curves of KNN methods to classification. (a) ROC curve of training set, the AUC were 0.988690981057 in NTM (sensitivity and specificity were 0.92 and {"TB": 0.92, "NTM": 0.96}), 0.988690981057 in TB (sensitivity and specificity were 0.96 and {"TB": 0.92, "NTM": 0.96}) respectively; (b) ROC curve of validation set,

the AUC were 0.987012987013 in NTM (sensitivity and specificity were 0.94 and {"TB": 0.94, "NTM": 0.96}), 0.987012987013 in TB (sensitivity and specificity were 0.96 and {"TB": 0.94, "NTM": 0.96}) respectively.

SVM paramsters: kernel(rbf), C(1), gamma(auto), class_weight(balanced), decision_function_shape(ovr), random_state(),

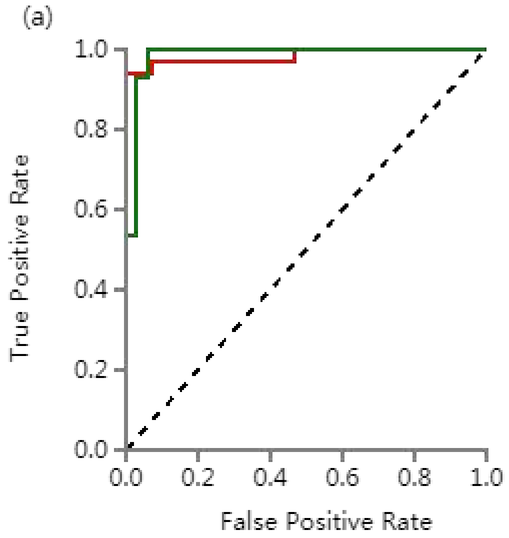

NTM(auc=0. 98) TB(auc=0. 98)

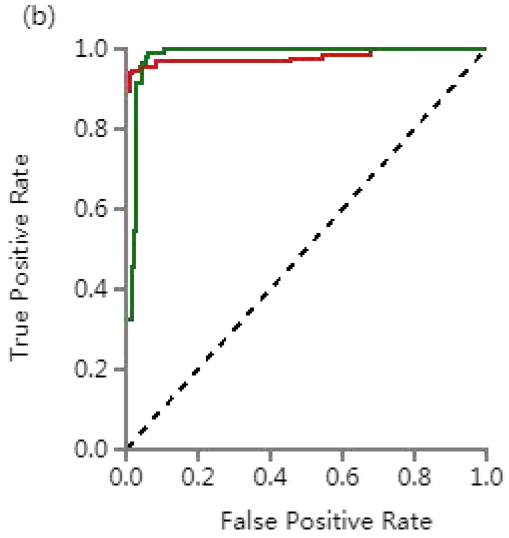

NTM(auc=0. 98) TB(auc=0. 98)

Fig. 5. ROC curves of SVM methods to classification. (a) ROC curve of training set, the AUC were 0.979926491377 in NTM (sensitivity and specificity were 0.95 and {"TB": 0.95, "NTM": 0.96}), 0.979926491377 in TB (sensitivity and specificity were

0.96 and {"TB": 0.95, "NTM": 0.96}) respectively; (b) ROC curve of validation set, the AUC were 0.983766233766 in NTM (sensitivity and specificity were 0.97 and {"TB": 0.97, "NTM": 0.82}), 0.983766233766 in TB (sensitivity and specificity were 0.82 and {"TB": 0.97, "NTM": 0.82}) respectively.

XGBoost paramsters: Eta(0.3), max_depth(6),

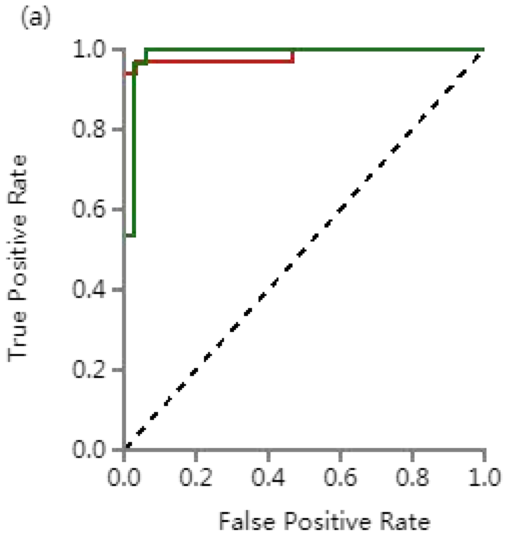

NTM(auc=0.98)TB(auc=0.98)

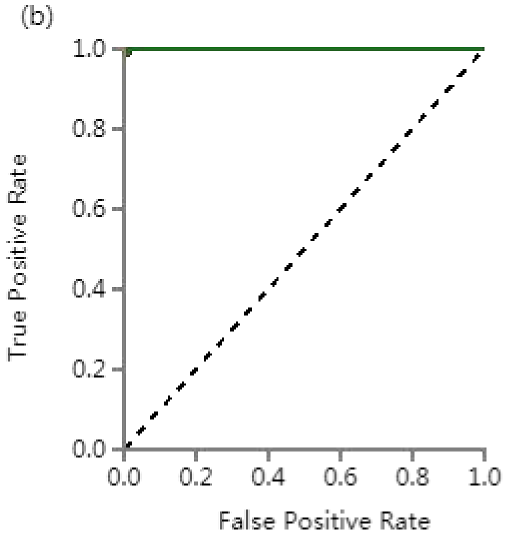

NTM(auc=0.98)TB(auc=0.98)

Fig. 6. ROC curves of XGBoost methods to classification. (a) ROC curve of training set, the AUC were 0.999858637263 in NTM (sensitivity and specificity were 0.98 and {"TB": 0.98, "NTM": 0.99}), 0.999858637263 in TB (sensitivity and specificity were

0.99 and {"TB": 0.98, "NTM": 0.99}) respectively; (b) ROC curve of validation set, the AUC were 0.984848484848 in NTM (sensitivity and specificity were 0.97 and {"TB": 0.97, "NTM": 0.96}), 0.984848484848 in TB (sensitivity and specificity were 0.96 and {"TB": 0.97, "NTM": 0.96}) respectively.

RF paramsters: n_estimators(10), class_weight(None),

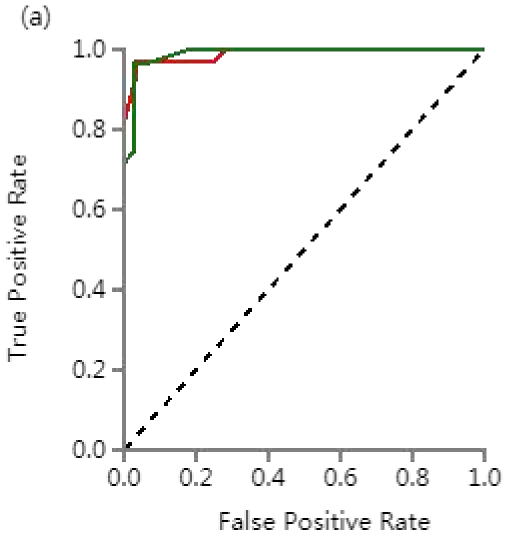

NTM(auc=0. 99) TB(auc=0. 99)

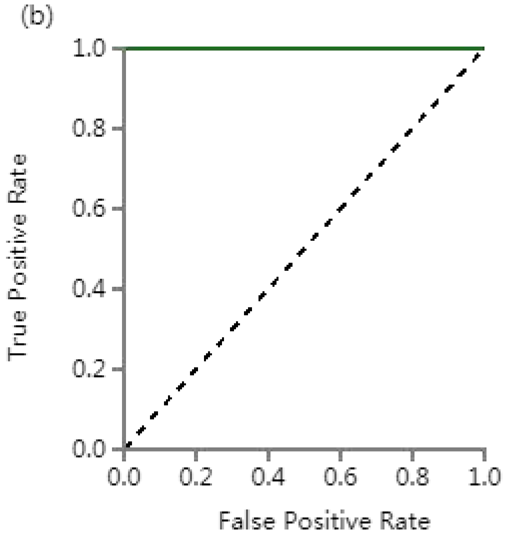

NTM(auc=0. 99) TB(auc=0. 99)

Fig. 7. ROC curves of RF methods to classification. (a) ROC curve of training set, the AUC were 0.999964659316 in NTM (sensitivity and specificity were 0.99 and {"TB": 0.99, "NTM": 1.0}), 0.999964659316 in TB (sensitivity and specificity were 1.00 and

{"TB": 0.99, "NTM": 1.0}) respectively; (b) ROC curve of validation set, the AUC were 0.988636363636 in NTM (sensitivity and specificity were 0.94 and {"TB": 0.94, "NTM": 0.96}), 0.988636363636 in TB (sensitivity and specificity were 0.96 and {"TB": 0.94, "NTM": 0.96}) respectively.

LR paramsters: penalty(L2), C(1), solver(liblinear), class_weight(None), multi_class(ovr), random_state(),

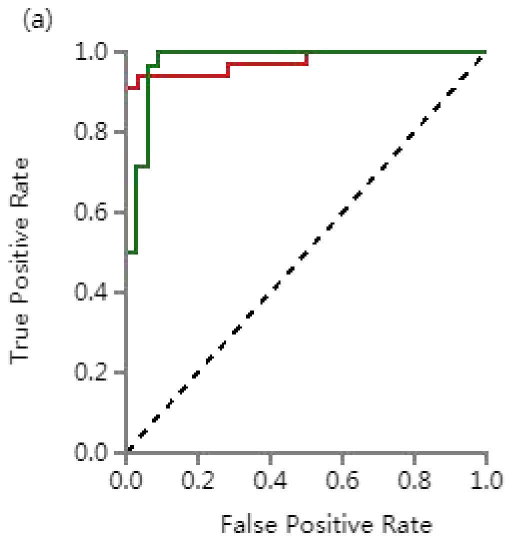

NTM(auc=0. 98) TB(auc=0. 98)

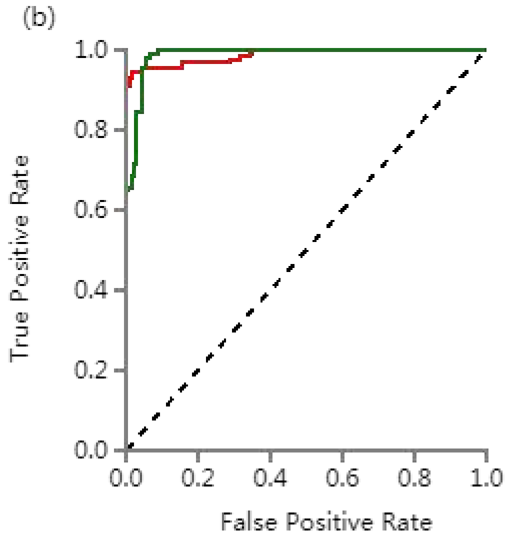

NTM(auc=0. 98) TB(auc=0. 98)

Fig. 8. ROC curves of LR methods to classification. (a) ROC curve of training set, the AUC were 0.986853265479 in NTM (sensitivity and specificity were 0.95 and {"TB":

0.95, "NTM": 0.95}), 0.986853265479 in TB (sensitivity and specificity were 0.95 and {"TB": 0.95, "NTM": 0.95}) respectively; (b) ROC curve of validation set, the AUC were 0.975108225108 in NTM (sensitivity and specificity were 0.94 and {"TB": 0.94, "NTM": 0.93}), 0.975108225108 in TB (sensitivity and specificity were 0.93 and {"TB": 0.94, "NTM": 0.93}) respectively.

DT paramsters: splitter(best), criterion(gini),

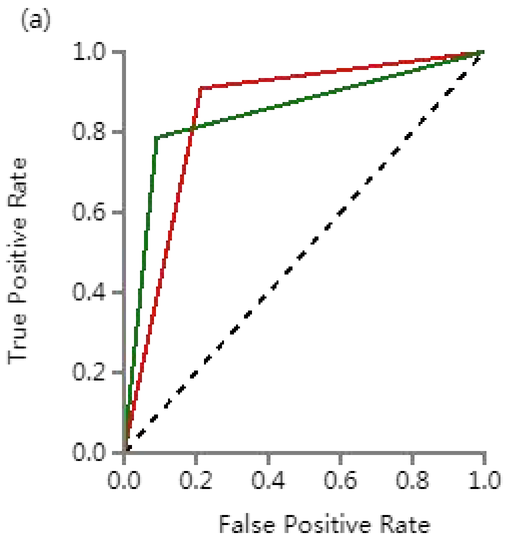

NTM(auc=0. 85) TB(auc=0. 85)

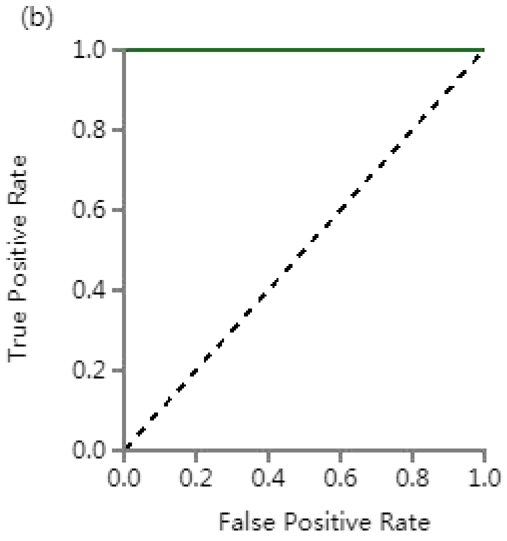

NTM(auc=0. 85) TB(auc=0. 85)

Fig. 9. ROC curves of DT methods to classification. (a) ROC curve of training set, the AUC were 1.0 in NTM (sensitivity and specificity were 1.00 and {"TB": 1.0, "NTM":

1.0}), 1.0 in TB (sensitivity and specificity were 1.00 and {"TB": 1.0, "NTM": 1.0}) respectively; (b) ROC curve of validation set, the AUC were 0.847402597403 in NTM (sensitivity and specificity were 0.91 and {"TB": 0.91, "NTM": 0.79}), 0.847402597403 in TB (sensitivity and specificity were 0.79 and {"TB": 0.91, "NTM": 0.79}) respectively.

4.References

1. Lambin P, Rios-Velazquez E, Leijenaar R, et al. Radiomics: extracting more information from medical images using advanced feature analysis. European journal of cancer, 2012, 48(4): 441-446.

2. Kumar V, Gu Y, Basu S, et al. Radiomics: the process and the challenges.[J]. Magnetic Resonance Imaging, 2012, 30(9): 1234-1248.

3. Gillies R J, Kinahan P E, Hricak H, et al. Radiomics: Images Are More than Pictures, They Are Data[J]. Radiology, 2016, 278(2): 563-577.

4. Wang YY, Zhang T, Li SWet al. Mapping p53 mutations in low-grade glioma: a voxel-based neuroimaging analysis. AJNR Am J Neuroradiol, 2015, 36:70–76.

Appendix

Radcloud cloud platform

Radiomics cloud platform uses cloud computing, big data analysis and machine learning algorithms to manage DICOM imaging data, medical check-up reports and clinical information on cloud platforms of hospitals. Anytime, anywhere, with only one click, research fellows can use the radiomics cloud platform to process and analyze data.

Research Project Management

Management of user access on data sharing among research project teams is now possible; separation of individual’s projects and cooperation projects is already a reality. This platform supports cloud management, remote cooperation and group sharing.

Multi-center Cooperation Management

Based on mixed cloud construction and user access management, this platform supports group visiting and cross-department, inter-agency cooperation.

Radiomics Statistics Analysis

More than 1,000 Ml eigenvalue for analysis, combined with machine learning, we have the one-dick operation for analysts.

Imaging and Clinical Data Management

Supports auto batch upload of imaging data, simultaneous data cleansing, input of dinical and pathological data. Also supports precise searching of Ml.
