# Supplementary material for: Differentiating nontuberculous mycobacterium pulmonary disease from pulmonary tuberculosis through the analysis of the cavity features in CT images using radiomics
Source: BMC Pulm Med. 2022 Jan 7;22:4. doi: 10.1186/s12890-021-01766-2 (PMC8740493; doi:10.1186/s12890-021-01766-2)
Supplement: Supplementary file 2 — Additional file 2. External validation of the model in this study. [file 12890_2021_1766_MOESM2_ESM.docx]

# 数据集

用于模型训练和内部验证的患者共142例，标注了300个voi（164个NTM，136个TB）

用于外部验证的患者**40例，标注了79个voi（49个NTM，30个TB）**

# 外部验证结果

共提取出1409个放射组学特征，对其中20例患者进行ICC检验，取p>0.75的特征进行下一步分析。其中p>0.75的特征有1367个，检验不符合要求的42个特征见表格“ICC_Coef_failed”。

## dt模型

### Validation_roc：


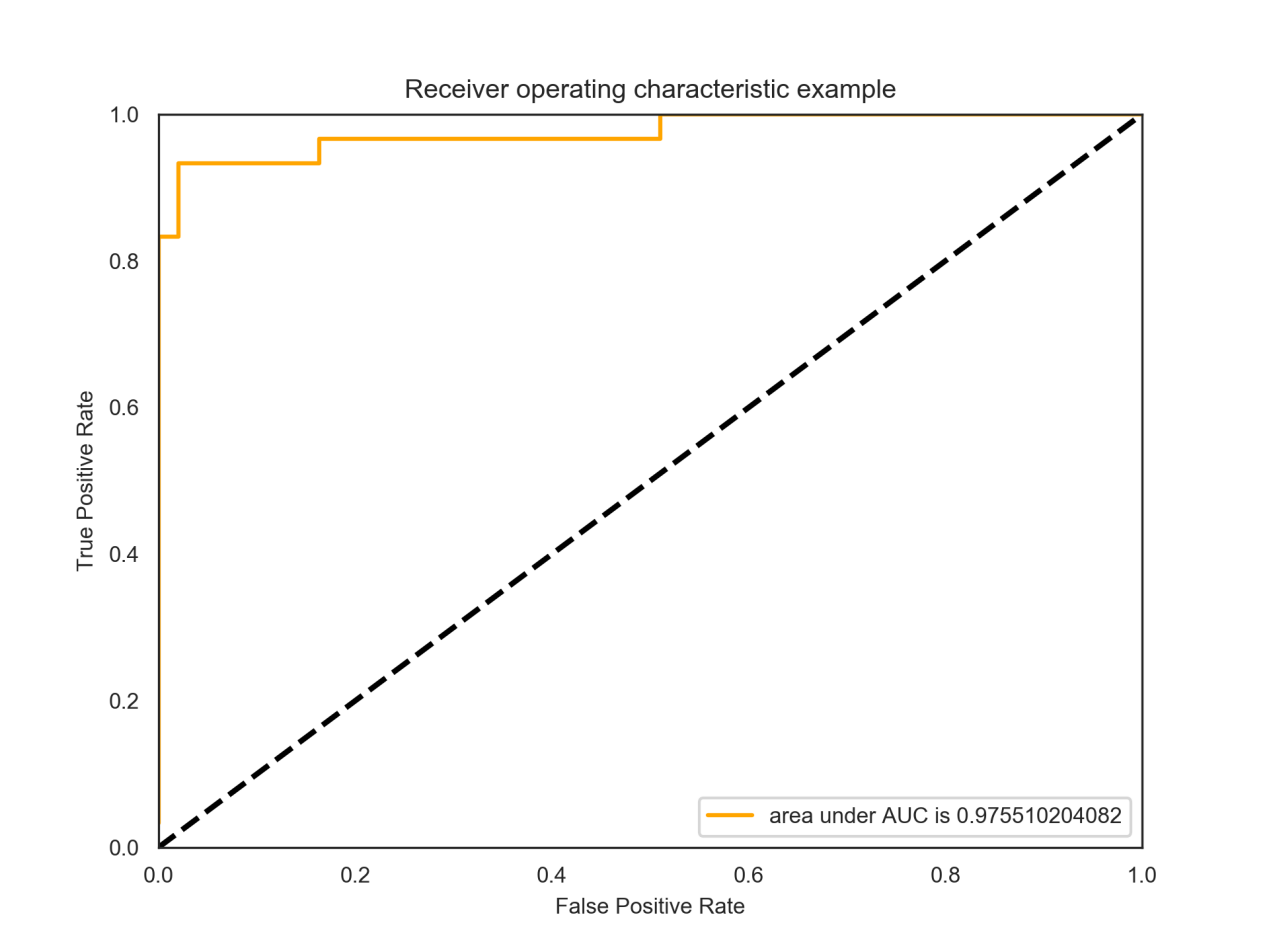


### 混淆矩阵：

|  | | 预测 | |
| --- | --- | --- | --- |
|  |  | NTM | TB |
| 实际 | NTM | 35 | 14 |
|  | TB | 1 | 29 |

评价指标：

Label precision recall f1-score support

NTM 0.97 0.71 0.82 49

TB 0.67 0.97 0.79 30

avg / total 0.86 0.81 0.81 79

| val_auc | val_acc(准确率) | val_sen(敏感度) | val_spe(特异度) | val_CI_L | val_CI_H |
| --- | --- | --- | --- | --- | --- |
| 0.975510204 | 0.810126582 | 0.714285714 | 0.966666667 | 0.775537634 | 0.894805195 |

## knn模型

### Validation_roc：


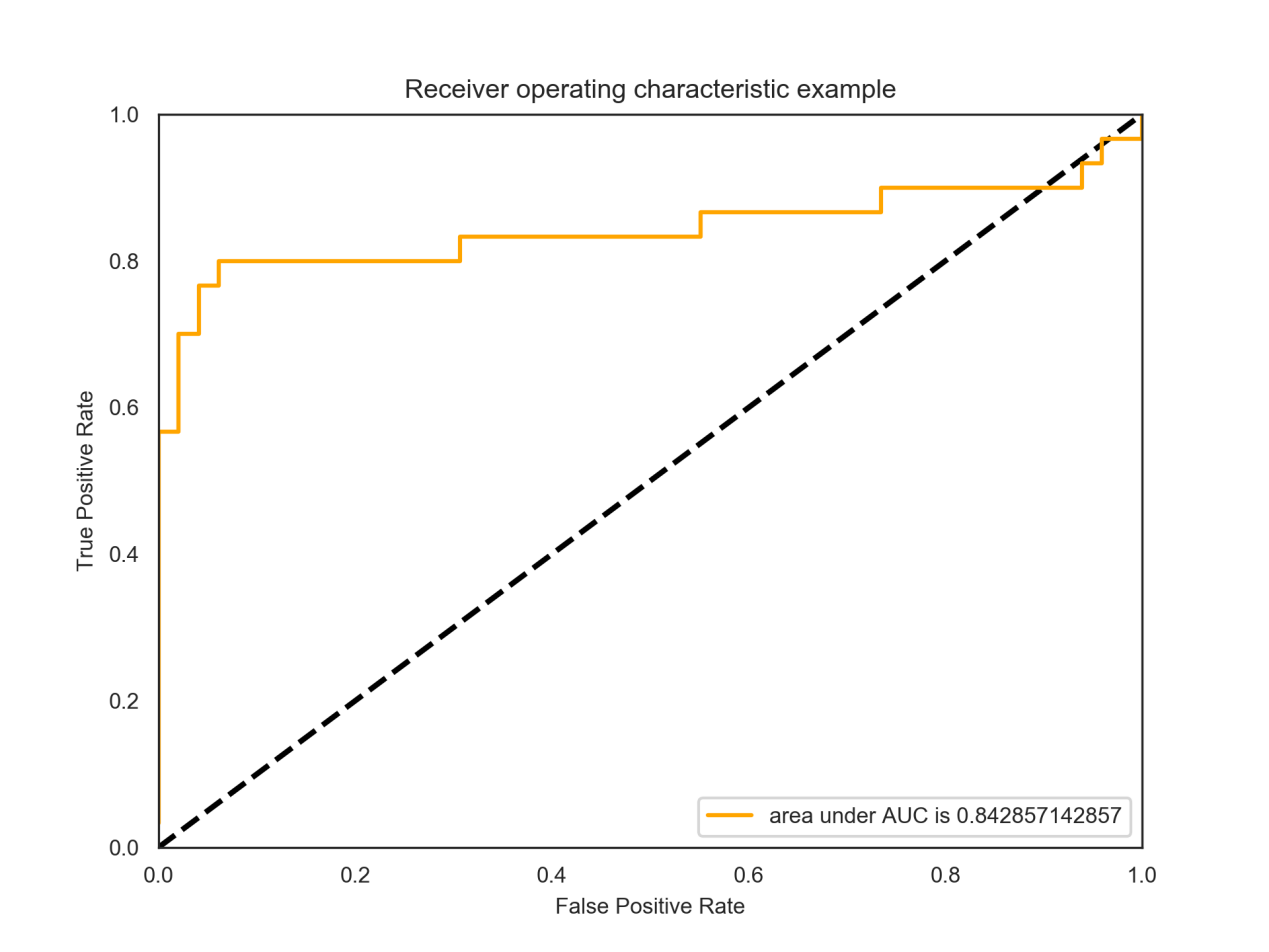


### 混淆矩阵：

|  | | 预测 | |
| --- | --- | --- | --- |
|  |  | NTM | TB |
| 实际 | NTM | 32 | 17 |
|  | TB | 5 | 25 |

评价指标：

Label precision recall f1-score support

NTM 0.86 0.65 0.74 49

TB 0.60 0.83 0.69 30

avg / total 0.76 0.72 0.73 79

| val_auc | val_acc(准确率) | val_sen(敏感度) | val_spe(特异度) | val_CI_L | val_CI_H |
| --- | --- | --- | --- | --- | --- |
| 0.842857143 | 0.721518987 | 0.653061224 | 0.833333333 | 0.65724734 | 0.819828722 |

## lr模型

### Validation_roc：


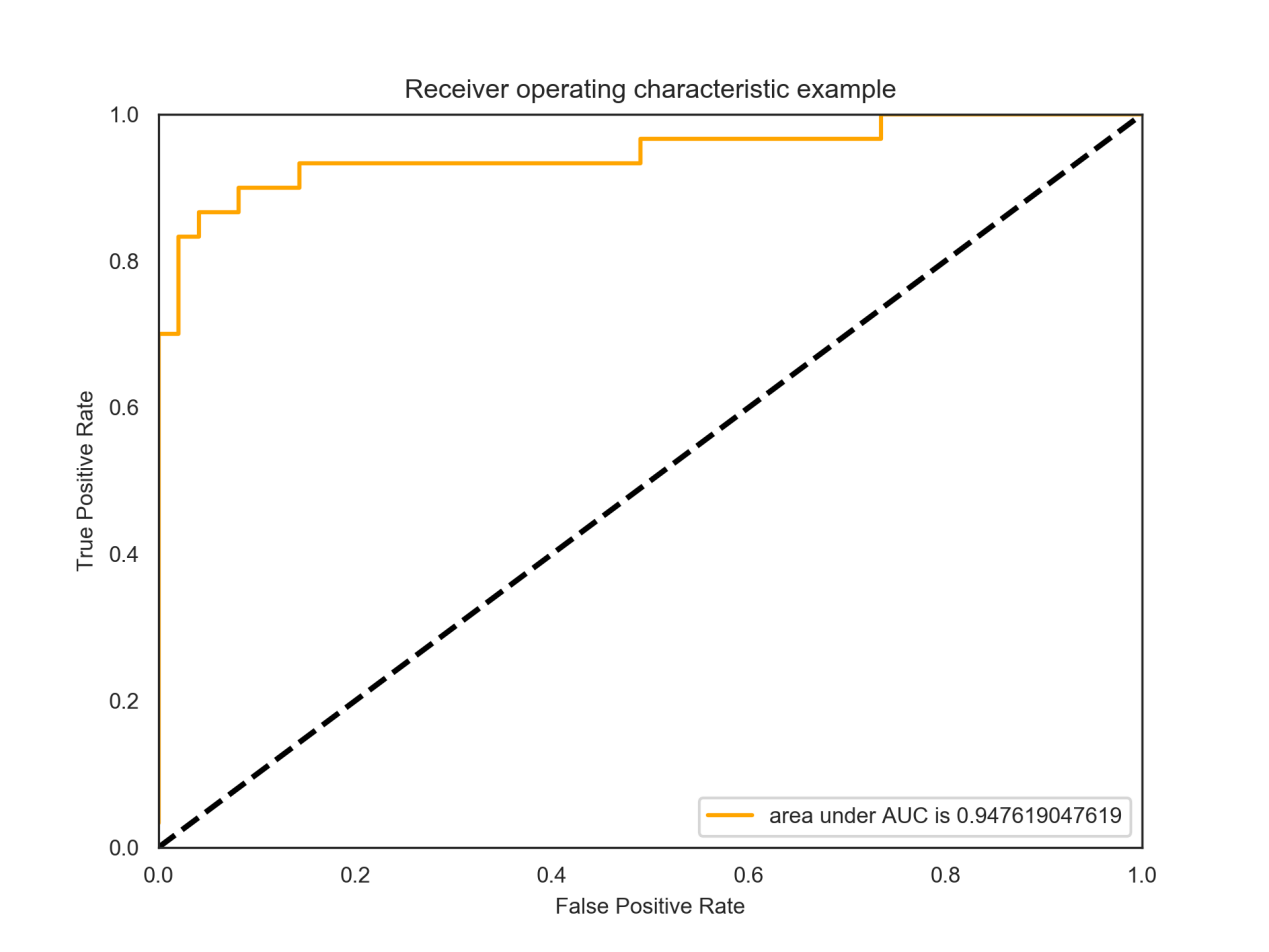


### 混淆矩阵：

|  | | 预测 | |
| --- | --- | --- | --- |
|  |  | NTM | TB |
| 实际 | NTM | 46 | 3 |
|  | TB | 4 | 26 |

评价指标：

Label precision recall f1-score support

NTM 0.92 0.94 0.93 49

TB 0.90 0.87 0.88 30

avg / total 0.91 0.91 0.91 79

| val_auc | val_acc(准确率) | val_sen(敏感度) | val_spe(特异度) | val_CI_L | val_CI_H |
| --- | --- | --- | --- | --- | --- |
| 0.947619048 | 0.911392405 | 0.93877551 | 0.866666667 | 0.841287879 | 0.956462585 |

## rf模型

### Validation_roc：


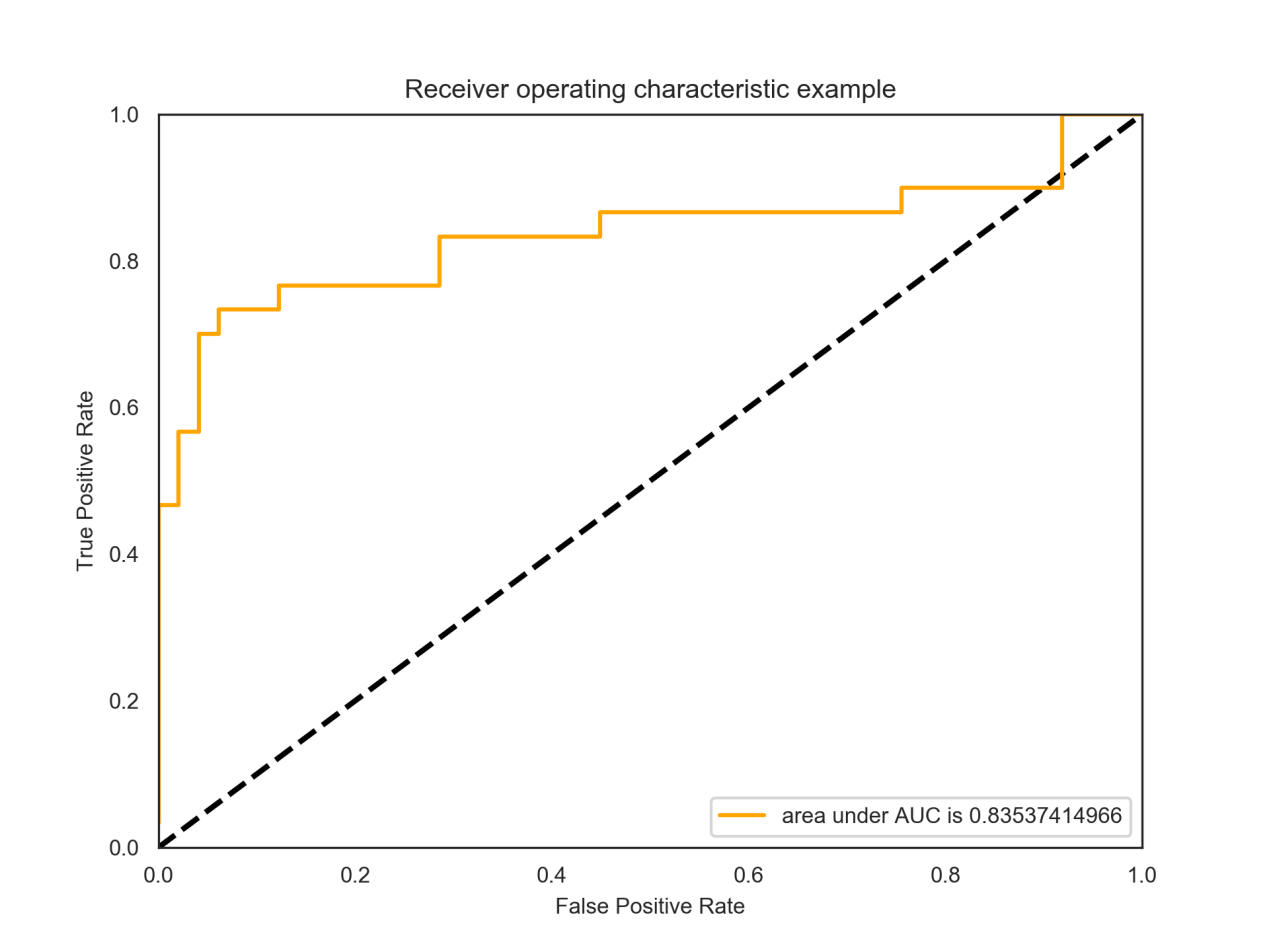


### 混淆矩阵：

|  | | 预测 | |
| --- | --- | --- | --- |
|  |  | NTM | TB |
| 实际 | NTM | 30 | 19 |
|  | TB | 5 | 25 |

评价指标：

Label precision recall f1-score support

NTM 0.86 0.61 0.71 49

TB 0.57 0.83 0.68 30

avg / total 0.75 0.70 0.70 79

| val_auc | val_acc(准确率) | val_sen(敏感度) | val_spe(特异度) | val_CI_L | val_CI_H |
| --- | --- | --- | --- | --- | --- |
| 0.83537415 | 0.696202532 | 0.612244898 | 0.833333333 | 0.635714286 | 0.796938776 |

## svm模型

### Validation_roc：


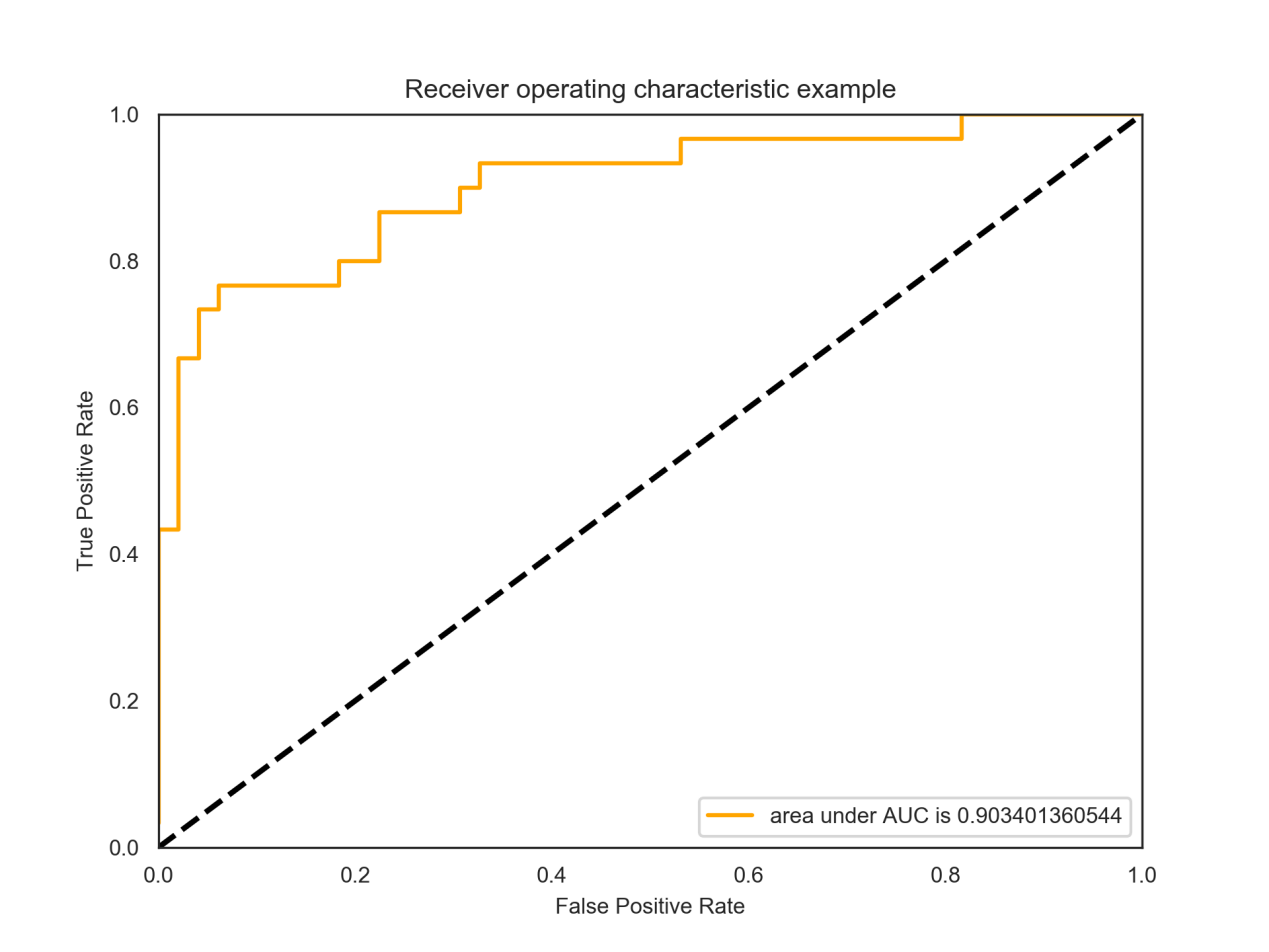


### 混淆矩阵：

|  | | 预测 | |
| --- | --- | --- | --- |
|  |  | NTM | TB |
| 实际 | NTM | 46 | 3 |
|  | TB | 7 | 23 |

评价指标：

Label precision recall f1-score support

NTM 0.87 0.94 0.90 49

TB 0.88 0.77 0.82 30

avg / total 0.87 0.87 0.87 79

| val_auc | val_acc(准确率) | val_sen(敏感度) | val_spe(特异度) | val_CI_L | val_CI_H |
| --- | --- | --- | --- | --- | --- |
| 0.903401361 | 0.873417722 | 0.93877551 | 0.766666667 | 0.777586207 | 0.917898194 |

## xgboost模型

### Validation_roc：


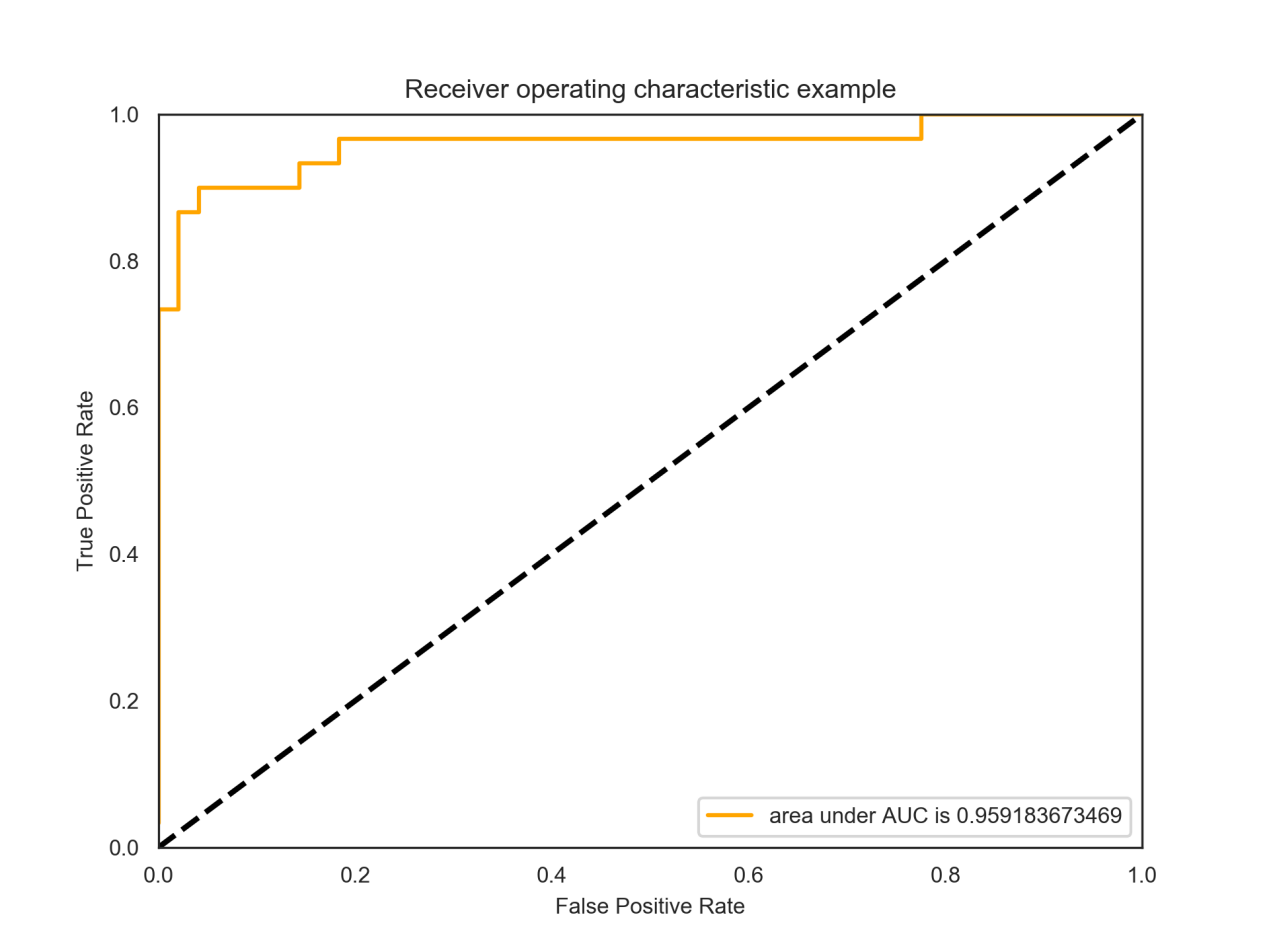


### 混淆矩阵：

|  | | 预测 | |
| --- | --- | --- | --- |
|  |  | NTM | TB |
| 实际 | NTM | 32 | 17 |
|  | TB | 1 | 29 |

评价指标：

Label precision recall f1-score support

NTM 0.97 0.65 0.78 49

TB 0.63 0.97 0.76 30

avg / total 0.84 0.77 0.77 79

| val_auc | val_acc(准确率) | val_sen(敏感度) | val_spe(特异度) | val_CI_L | val_CI_H |
| --- | --- | --- | --- | --- | --- |
| 0.959183673 | 0.772151899 | 0.653061224 | 0.966666667 | 0.748275862 | 0.869047619 |
