# Supplementary figures and images for: Differentiating nontuberculous mycobacterium pulmonary disease from pulmonary tuberculosis through the analysis of the cavity features in CT images using radiomics
Source: BMC Pulm Med. 2022 Jan 7;22:4. doi: 10.1186/s12890-021-01766-2 (PMC8740493; doi:10.1186/s12890-021-01766-2)

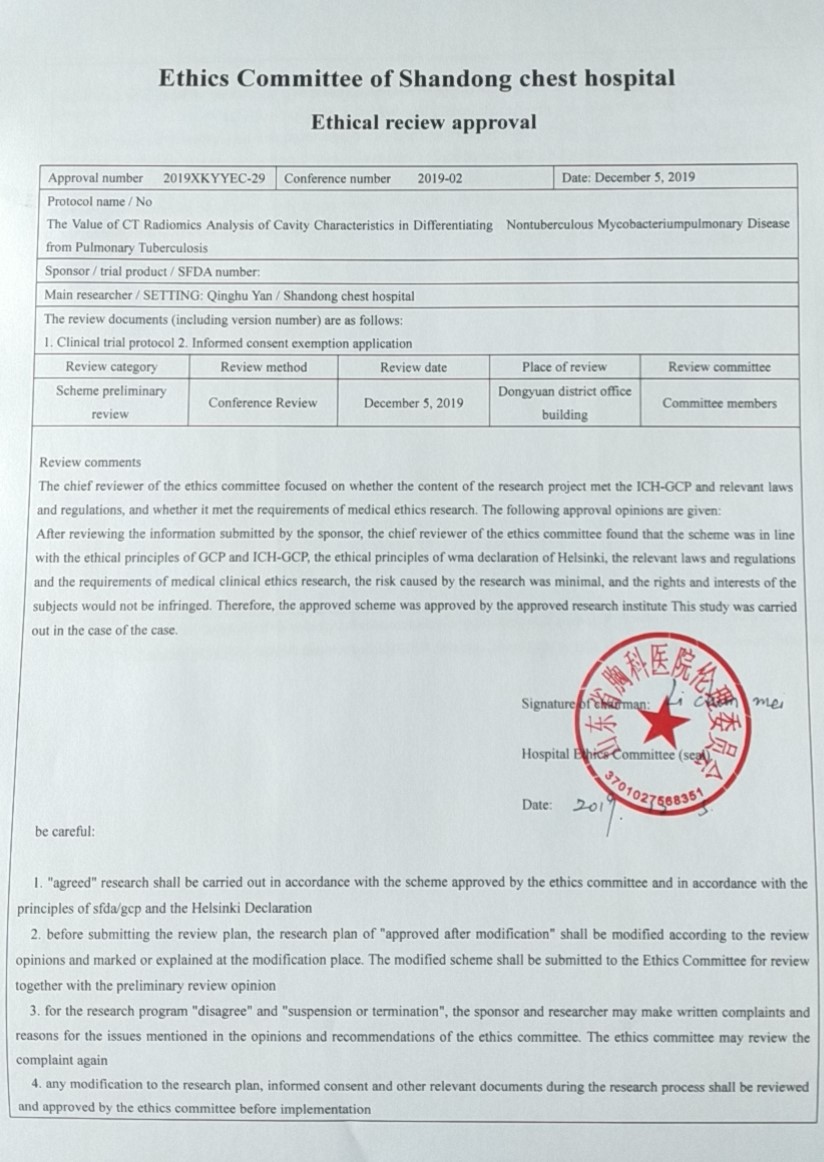

Supplement: Supplementary file 3 — Additional file 3. Ethical proof. [file 12890_2021_1766_MOESM3_ESM.jpg]
